# Porcine Feed Efficiency-Associated Intestinal Microbiota and Physiological Traits: Finding Consistent Cross-Locational Biomarkers for Residual Feed Intake

**DOI:** 10.1128/mSystems.00324-18

**Published:** 2019-06-18

**Authors:** Ursula M. McCormack, Tânia Curião, Barbara U. Metzler-Zebeli, Elizabeth Magowan, Donagh P. Berry, Henry Reyer, Maria L. Prieto, Stefan G. Buzoianu, Michael Harrison, Natalie Rebeiz, Fiona Crispie, Paul D. Cotter, Orla O’Sullivan, Gillian E. Gardiner, Peadar G. Lawlor

**Affiliations:** a Teagasc, Pig Development Department, Animal and Grassland Research and Innovation Centre, Moorepark, Fermoy, Co. Cork, Ireland; b Department of Science, Waterford Institute of Technology, Waterford, Co. Waterford, Ireland; c Institute of Animal Nutrition and Functional Plant Compounds, University Clinic for Swine, University of Veterinary Medicine Vienna, Vienna, Austria; d Sustainable Agri-Food Sciences Division, Agri-Food and Biosciences Institute, Hillsborough, Co. Down, Northern Ireland; e Teagasc, Animal and Bioscience Research Department, Animal and Grassland Research Centre, Moorepark, Fermoy, Co. Cork, Ireland; f Leibniz Institute for Farm Animal Biology, Dummerstorf, Germany; g Teagasc, Food Research Centre, Moorepark, Fermoy, Co. Cork, Ireland; h APC Microbiome Institute, Cork, Ireland; Pacific Northwest National Laboratory

**Keywords:** feed efficiency, geographic location, intestinal microbiota, pigs

## Abstract

Optimal feed efficiency (FE) in pigs is important for economic and environmental reasons. Previous research identified FE-associated bacterial taxa within the intestinal microbiota of growing pigs. This study investigated whether FE-associated bacteria and selected FE-associated physiological traits were consistent across geographic locations (Republic of Ireland [ROI] [two batches of pigs, ROI1 and ROI2], Northern Ireland [NI], and Austria [AT]), where differences in genetic, dietary, and management factors were minimized. Pigs (*n* = 369) were ranked, within litter, on divergence in residual feed intake (RFI), and 100 extremes were selected (50 with high RFI and 50 with low RFI) across geographic locations for intestinal microbiota analysis using 16S rRNA amplicon sequencing and examination of FE-associated physiological parameters. Microbial diversity varied by geographic location and intestinal sampling site but not by RFI rank, except in ROI2, where more-feed-efficient pigs had greater ileal and cecal diversity. Although none of the 188 RFI-associated taxonomic differences found were common to all locations/batches, *Lentisphaerae*, *Ruminococcaceae*, RF16, *Mucispirillum*, *Methanobrevibacter*, and two uncultured genera were more abundant within the fecal or cecal microbiota of low-RFI pigs in two geographic locations and/or in both ROI batches. These are major contributors to carbohydrate metabolism, which was reflected in functional predictions. Fecal volatile fatty acids and salivary cortisol were the only physiological parameters that differed between RFI ranks. Despite controlling genetics, diet specification, dietary phases, and management practices in each rearing environment, the rearing environment, encompassing maternal influence, herd health status, as well as other factors, appears to impact intestinal microbiota more than FE.

**IMPORTANCE** Interest in the role of intestinal microbiota in determining FE in pigs has increased in recent years. However, it is not known if the same FE-associated bacteria are found across different rearing environments. In this study, geographic location and intestinal sampling site had a greater influence on the pig gut microbiome than FE. This presents challenges when aiming to identify consistent reliable microbial biomarkers for FE. Nonetheless, seven FE-associated microbial taxa were common across two geographic locations and/or two batches within one location, and these indicated a potentially “healthier” and metabolically more capable microbiota in more-feed-efficient pigs. These taxa could potentially be employed as biomarkers for FE, although bacterial consortia, rather than individual taxa, may be more likely to predict FE. They may also merit consideration for use as probiotics or could be targeted by dietary means as a strategy for improving FE in pigs in the future.

## Abstract (precis)

Interest in the role of intestinal microbiota in determining FE in pigs has increased in recent years. However, it is not known if the same FE-associated bacteria are found across different rearing environments. In this study, geographic location and intestinal sampling site had a greater influence on the pig gut microbiome than FE. This presents challenges when aiming to identify consistent reliable microbial biomarkers for FE. Nonetheless, seven FE-associated microbial taxa were common across two geographic locations and/or two batches within one location, and these indicated a potentially “healthier” and metabolically more capable microbiota in more-feed-efficient pigs. These taxa could potentially be employed as biomarkers for FE, although bacterial consortia, rather than individual taxa, may be more likely to predict FE. They may also merit consideration for use as probiotics or could be targeted by dietary means as a strategy for improving FE in pigs in the future.

## INTRODUCTION

Feed efficiency (FE) is critical in pig production, as feed accounts for ∼70% of production costs (1). As a result, a considerable amount of research has focused on improving FE and finding reliable biomarkers for FE in pigs. Those that have been suggested include cortisol, a hormone which is secreted in response to stress and low blood glucose levels, affecting metabolism of carbohydrates, lipids, and protein (2). Animals with a higher level of blood plasma cortisol are more likely to divert energy away from lean-meat deposition, resulting in poorer FE (3). Blood cell profile and metabolic markers are functional indicators of metabolic pathways that ultimately denote homeostasis or dysfunction (4), and some of these have also been linked with FE in pigs (e.g., hemoglobin concentration; red blood cell count; white blood cell profile; and protein, triglyceride, and cholesterol concentrations) (5–7). Immune status is another important factor associated with FE; it has been suggested that pigs with enhanced FE might have a more effective immune response without affecting growth and lean-meat deposition (8, 9). More-feed-efficient pigs were previously shown to produce lower but sufficient levels of white blood cells, i.e., lymphocytes and monocytes (5), and had increased expression of antigen-processing-related genes (10).

The resident intestinal microbiota, specifically its diversity, composition, and function, is likely to influence FE in pigs, considering its role in host metabolism and immunity (9, 11–13). An increasing number of studies aimed at characterizing the swine intestinal microbiome have identified major populations of bacteria associated with intestinal site, age of the pig, and diet (14–19). Studies have also uncovered a major role for the porcine intestinal microbiota and microbial metabolites in regulation of the immune system (20). Recently, a number of studies, including one from our group, have demonstrated an association between porcine intestinal microbiota composition and FE (12, 13, 19, 21). However, each study was limited to one batch of pigs reared in the same environment at a single geographic location.

Humans living in different environments have dramatically different intestinal microbial profiles (22), whereas in cattle, management practices appear to be more influential than geographic location (23). Previous work by this group investigated the fecal and intestinal microbiota of pigs divergent in RFI (residual feed intake; a metric for FE) within one rearing environment (12). Feed efficiency-associated bacteria were identified throughout the lifetime of the pig albeit mostly low-relative-abundance taxa. In general, the abundances of microbes related to metabolism and disease were higher and lower, respectively, in low-RFI (more-feed-efficient) pigs. Following on from this work, where a first set of FE-associated microbial taxa was identified, the present study sought to determine if these findings can be extrapolated to pigs raised in different rearing environments, even other countries, when genetics, diet, and management are controlled.

The objective was to investigate the intestinal microbiota of pigs ranked on RFI, reared at three geographic locations, each using the same animal genetics, diet specifications, dietary phases, and management protocols, with a view to determining if similar FE-associated microbiomes are found across different rearing environments. Other physiological parameters were also assessed and correlated with intestinal microbial composition, in an attempt to elucidate their role in influencing FE in pigs.

## RESULTS

### Growth performance of pigs ranked as having low or high RFI at different geographic locations.

Growth performance parameters, including residual feed intake (RFI), were determined between days 70 and 120 of age and are presented in Table 1. Across geographic locations (Republic of Ireland [ROI], Northern Ireland [NI], and Austria [AT]), there was a distinct separation between high- and low-RFI pigs (*P* < 0.001). The average daily gain (ADG) (*P* = 0.25) and weight at day 70 (*P* = 0.22) did not differ between RFI ranks across locations, but weight at day 120 (*P* < 0.001), average daily feed intake (ADFI) (*P* < 0.005), and feed conversion efficiency (FCE) (*P* < 0.03) did.

**TABLE 1:** Growth performance and salivary cortisol^*a*^ of pigs ranked on residual feed intake at three geographic locations^*b*^

Parameter	Value for group^*c*^								SEM^*d*^	*P* value		
	High RFI				Low RFI							
	ROI1	ROI2	NI	AT	ROI1	ROI2	NI	AT		RFI × location	RFI	Location
RFI (g/day)	1,237 A	1,108 A	207 B	1,171 AB	−1,030 C	−737 C	−200 C	−956 C	227.6	<0.001	<0.001	0.86
wt (kg), day 70	32.2	33.3	27.4	30.2	33.6	31.9	28.7	28.6	1.93	0.22	0.97	0.27
wt (kg), day 134	83.1 BC	82.3 BC	91.3 AC	85.2 ABC	87.2 ABC	79.5 B	95.2 B	82.8 BC	1.94	<0.001	0.83	0.35
ADFI (g/day)	2,025 BD	2,194 AD	2,033 AB	2,380 A	1,988 BD	1,851 BC	1,586 C	1,942 BCD	74.1	0.005	<0.001	0.005
ADG (g/day)	1,028	902	891	1,131	1,065	847	894	1,077	30.0	0.25	0.44	<0.001
FCE (g/g)	2.05 B	2.62 A	2.40 A	2.11 B	1.91 B	2.39 A	1.80 B	1.84 B	0.088	0.03	<0.001	<0.001

Salivary cortisol concn (ng/ml)	1.76				1.34				0.176		0.06	

a Cortisol was measured only in ROI1 pigs on the day prior to slaughter.

b ROI1, Republic of Ireland batch 1; ROI2, Republic of Ireland batch 2; NI, Northern Ireland; AT, Austria; wt, weight; ADFI, average daily feed intake; ADG, average daily gain; FCE, feed conversion efficiency.

c Within each row, values that do not share a common letter are different (*P* ≤ 0.05).

d Least-squares means and the pooled standard errors of the means (SEM) are presented.

### Microbial diversity and composition in the feces and digesta of pigs ranked on RFI at different geographic locations.

**FIG 1:**
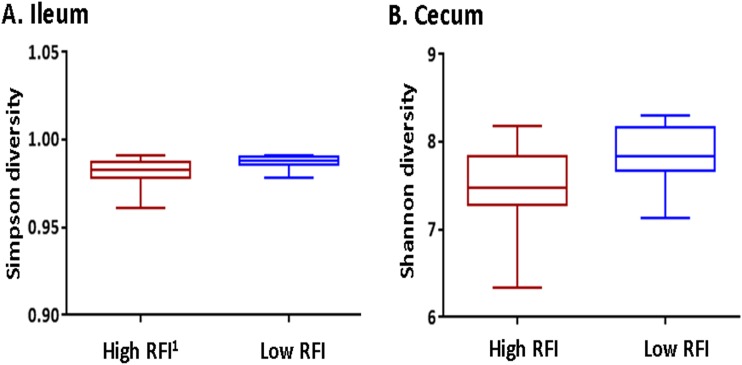
Alpha diversity of the microbiota within the ileum (A) and cecum (B) of pigs ranked on residual feed intake (RFI^1^) from Republic of Ireland batch 2 (ROI2).

Microbial richness and diversity within the feces of pigs (at both days 70 and 134 of age) were not associated with RFI rank (see Fig. S1 in the supplemental material). However, in the ileal and cecal digesta from ROI batch 2 (ROI2), operational taxonomic unit (OTU) diversity, depicted by Shannon and Simpson α-diversity indices, was higher for low-RFI pigs (*P* < 0.05) (Fig. 1).

10.1128/mSystems.00324-18.1 FIG S1 Alpha diversity of the fecal microbiota of pigs ranked on residual feed intake (RFI) at 70 days of age (A) and 134 days of age (B) across all three geographic locations. ROI, Republic of Ireland; NI, Northern Ireland; AT, Austria. Download FIG S1, DOCX file, 0.3 MB. Copyright © 2019 McCormack et al. 2019 McCormack et al. This content is distributed under the terms of the Creative Commons Attribution 4.0 International license.

**FIG 2:**
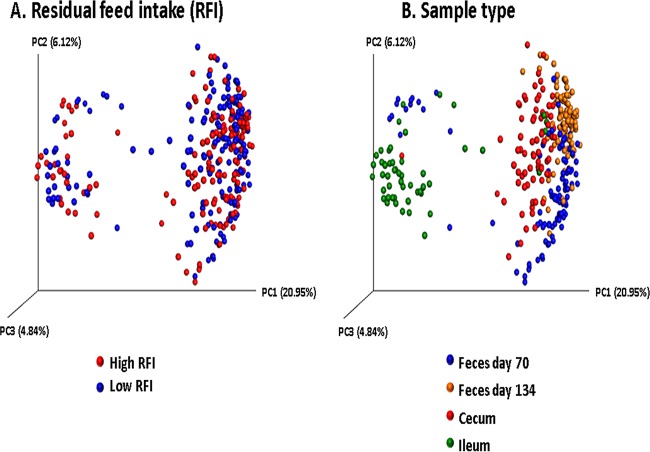
Principal-coordinate analysis (PCoA) plots (based on OTUs) by RFI rank (A) and sample type (B).

**FIG 3:**
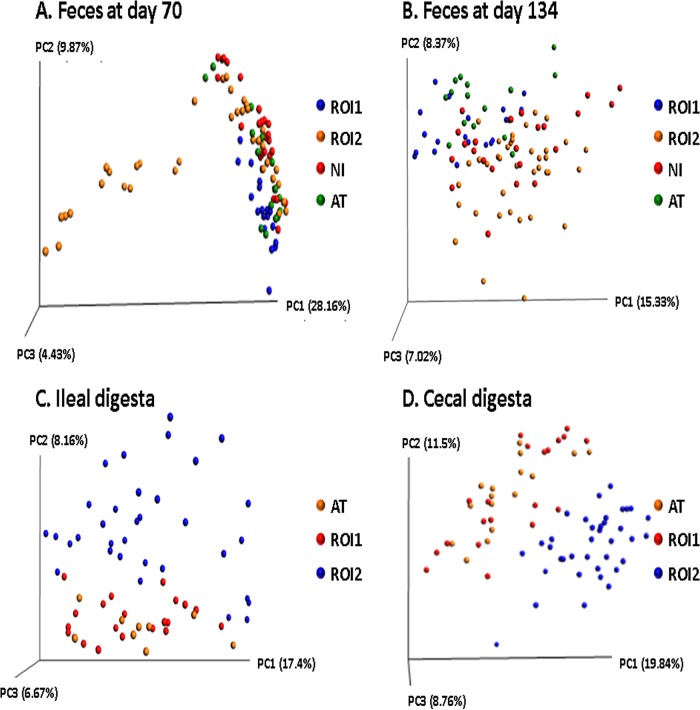
Principal-coordinate analysis plots (based on OTUs) for feces collected from pigs on day 70 (A) and day 134 (B) of age and in ileal (C) and cecal (D) digesta. ROI, Republic of Ireland; AT, Austria; NI, Northern Ireland.

Overall, β-diversity of the intestinal microbiota was affected by sample type (i.e., feces and/or digesta) and age at sampling but not by RFI rank (Fig. 2). At each fecal time point and for each digesta type, location-specific effects were observed, with samples from the same geographic location/digesta type generally clustering together in the principal-coordinate analysis (PCoA) plots (*P* < 0.001) (Fig. 3). However, one exception was that samples from ROI1 and ROI2 pigs did not cluster together, even though they originated from the same location. In fact, the microbial diversity of samples from ROI1 pigs was closer to that of samples from AT pigs, and the samples from pigs from ROI2 were more similar to samples from NI pigs.

Microbial composition was examined, at the phylum, family, and genus levels. The phylum *Firmicutes* was the most abundant phylum in the feces and ileal digesta in pigs from all geographic locations (Fig. S2). Pigs from ROI1 had a greater abundance of *Tenericutes* in the ileum than did pigs in ROI2 and AT (*P* < 0.05) (Fig. S2).

10.1128/mSystems.00324-18.2 FIG S2 Median relative abundance (percent) of bacterial phyla present in pigs according to residual feed intake (RFI) rank across geographic locations for all fecal time points and intestinal sites. ^1^Other (no BLAST hits/uncultured). * indicates a significant difference observed for that phylum between high- and low-RFI pigs within each sample type and geographic location (*P* ≤ 0.05). These RFI-associated differences are as follows: for feces on day 70, ↑ *Deferribacteres* (AT), ↑ SHA.109 (ROI2), and ↓ *Verrucomicrobia* (ROI1); for feces on day 134, ↓ candidate division TM7 (ROI2), ↓ *Firmicutes* (ROI1), ↑ *Euryarchaeota* (ROI1), and ↑ *Lentisphaerae* (ROI1 and ROI2) and *Verrucomicrobia* (ROI1); for ileum, ↓ *Cyanobacteria* (ROI2), ↓ *Firmicutes* (ROI2), and ↑ *Planctomycetes* (ROI2); and for cecum, ↑ *Cyanobacteria* (AT), ↓ *Firmicutes* (ROI1), ↑ *Proteobacteria* (ROI1), and ↑ *Verrucomicrobia* (ROI2). Arrows indicate higher (↑) or lower (↓) relative abundances in low-RFI pigs. Download FIG S2, DOCX file, 0.2 MB. Copyright © 2019 McCormack et al. 2019 McCormack et al. This content is distributed under the terms of the Creative Commons Attribution 4.0 International license.

When the intestinal microbiota of high- versus low-RFI pigs was compared at each geographic location at the phylum, family, and genus levels, a total of 188 compositional differences, mostly for taxa at a low relative abundance, were found across all locations (*P* < 0.05) (Tables 2 and 3). Most of these RFI-associated differences were observed in ROI2 pigs. Phylum-level differences between high- and low-RFI pigs are shown in Fig. S2 and included the high-abundance phylum *Firmicutes.* A number of low-relative-abundance phyla also differed significantly between RFI ranks, including *Proteobacteria*, *Verrucomicrobia*, SHA.109, *Deferribacteres*, *Lentisphaerae*, *Euryarchaeota*, candidate division TM7, *Cyanobacteria*, and *Planctomycetes*. For the majority of phyla, a higher relative abundance was observed in the low-RFI (i.e., more-feed-efficient) pigs than in their high-RFI counterparts, except for *Firmicutes* and *Verrucomicrobia* in ROI1 at day 70 of age.

**TABLE 2:** RFI-associated statistically significant microbial composition differences in the feces of pigs at three geographic locations^*a*^

Time point	Phylum^*b*^	Family	Genus	Location(s)^*c*^	Significance^*d*^	Difference^*e*^
Day 70 of age	*Bacteroidetes*	S24.7	Uncultured	NI	F, G	↓
				AT	G	↑
	*Deferribacteres*	*Deferribacteraceae*	Mucispirillum schaedleri	AT	P, G	↑
			*Mucispirillum*	NI	F, G	↑
				AT	G	↑

Day 134 of age	*Firmicutes*	Family XIII	*Mogibacterium*	NI	G	↓
		*Lachnospiraceae*	*Roseburia*	NI	G	↑
		*Ruminococcaceae*	*Incertae sedis*	ROI1	G	↓
			Uncultured	NI	G	↓
		*Erysipelotrichaceae*	Uncultured	NI	G	↓
	*Planctomycetes*	*Planctomycetaceae*		ROI2	F	↑
	SHA.109	Uncultured	Uncultured	ROI2	P, F, G	↑
	*Spirochaetes*	*Spirochaetaceae*	Treponema brennaborense	AT	G	↑
	*Verrucomicrobia*			ROI1	P	↓
				ROI1	P	**↑**
	*Euryarchaeota*	*Methanobacteriaceae*		AT	F	↑
		*Methanobrevibacter*		ROI1, ROI2	G	↑
		BS11.gut.group	Uncultured	ROI2	F, G	↑
		*Prevotellaceae*	*Alloprevotella*	ROI2	G	↓
	*Bacteroidetes*	RF16	Uncultured	ROI1	F, G	↑
				AT	G	↑
		*Rikenellaceae*	*Alistipes*	ROI2	G	↑
		Uncultured	Uncultured	ROI1	F, G	↓
	Candidate division TM7	Unknown family	“*Candidatus* Saccharimonas”	ROI2	P, F, G	↓
	*Cyanobacteria*	Uncultured	Uncultured	ROI1	F, G	↑
	*Firmicutes*	*Defluviitaleaceae*	Uncultured	ROI1	P, F, G	↓
		Family XI	*Sedimentibacter*	ROI2	F, G	↑
		Family XIII	Uncultured	ROI2	F, G	↑
		*Lachnospiraceae*	*Lachnospira*	ROI1	G	↓
			*Marvinbryantia*	ROI2	G	↓
			*Oribacterium*	NI	G	↑
				ROI1	G	↓
			*Pseudobutyrivibrio*	NI	G	↑
		*Ruminococcaceae*	*Faecalibacterium*	ROI2	G	↓
			*Papillibacter*	ROI2	G	↑
			*Ruminococcus*	NI	G	↓
			*Subdoligranulum*	ROI2	G	↓
		vadinBB60	Unidentified	ROI2	F, G	↑
		Uncultured	Unidentified	AT	G	↑
		*Erysipelotrichaceae*	*Asteroleplasma*	ROI2	G	↓
			*Incertae sedis*	ROI1	G	↓
		*Veillonellaceae*	*Allisonella*	ROI1	G	↓
			*Mitsuokella*	ROI1	G	↓
			*Selenomonas*	ROI2	G	↓
			Uncultured	ROI2	G	↓
				ROI1, ROI2	P	↑
	*Lentisphaerae*	RF12 gut group, uncultured	Uncultured	ROI1	F, G	↑
			Uncultured rumen bacterium	ROI1	F, G	↑
		RF12 gut group, unidentified	Uncultured	ROI1	F, G	↑
		WCHB1.25, uncultured	Uncultured	ROI1	F, G	↑
	*Proteobacteria*	*Neisseriaceae*	*Leeia*	ROI1	F, G	↑
		*Succinivibrionaceae*	Uncultured	ROI1	G	↑
		TA18, uncultured	Uncultured	ROI2	F, G	↑
		*Desulfovibrionaceae*	*Desulfovibrio*	AT	F, G	↑
	*Tenericutes*	RF9, uncultured	Uncultured	AT	F, G	↑
	*Verrucomicrobia*	*Verrucomicrobiaceae*	*Akkermansia*	ROI1	P, F, G	↑

a A total of 84 RFI-associated microbial composition differences were observed in the feces across the three geographic locations: 9 phyla, 23 families, and 52 genera.

b All phylum-level differences between high- and low-RFI pigs are shown in Fig. S2 in the supplemental material.

c AT, Austria; NI, Northern Ireland; ROI, Republic of Ireland (batches 1 and 2).

d Significantly different (*P* < 0.05) at the phylum (P), family (F), and/or genus (G) level.

e Arrows indicate relative-abundance differences in low-RFI pigs compared to high-RFI pigs at the same geographic location (↓, lower relative abundance; ↑, higher relative abundance).

**TABLE 3:** RFI-associated statistically significant microbial composition differences in the ileal and cecal digesta of pigs slaughtered at 134 days of age at three geographic locations^*a*^

Sample type	Phylum^*b*^	Family	Genus	Location(s)^*c*^	Significance^*d*^	Difference^*e*^
Ileum	*Actinobacteria*	*Dietziaceae*	*Dietzia*	ROI2	F, G	↑
		*Microbacteriaceae*	*Leucobacter*	ROI2	G	↑
		*Micrococcaceae*	*Rothia*	ROI2	G	↑
		*Pseudonocardiaceae*	*Saccharopolyspora*	ROI2	F, G	↑
	*Bacteroidetes*	S24.7	Uncultured	ROI2	G	↑
	*Cyanobacteria*	*Gastranaerophilales*, uncultured	Uncultured	ROI2	P, F, G	↑
	*Firmicutes*			ROI2	P	↓
		*Bacillaceae*	*Oceanobacillus*	ROI2	G	↑
			*Paucisalibacillus*	ROI2	G	↑
		*Paenibacillaceae*		ROI2	F	↑
		*Aerococcaceae*		ROI2	F	↑
			*Dolosicoccus*	ROI2	G	↑
			*Globicatella*	ROI2	G	↑
		*Enterococcaceae*	*Enterococcus*	ROI2	F, G	↑
		*Clostridiaceae* 1	*Clostridium sensu stricto* 1	ROI1	F, G	↑
		*Lachnospiraceae*	*Blautia*	ROI2	G	↑
			*Pseudobutyrivibrio*	ROI2	G	↑
			*Subdoligranulum*	ROI2	G	↑
		vadinBB60	Uncultured	ROI1	G	↓
				ROI2	G	↑
		*Erysipelotrichaceae*	*Turicibacter*	ROI1	F, G	↑
			*Solobacterium*	ROI2	G	↑
		*Veillonellaceae*	*Dialister*	ROI2	G	↑
			*Mitsuokella*	ROI2	G	↑
	*Planctomycetes*	*Planctomycetaceae*	p1088 a5 gut group	ROI2	P, F, G	↑
	*Proteobacteria*	*Desulfovibrionaceae*	*Desulfovibrio*	ROI2	F, G	↑
		*Helicobacteraceae*	*Helicobacter*	ROI1	F, G	↓
		*Pasteurellaceae*	*Actinobacillus*	ROI2	F, G	↑
	*Spirochaetae*	*Spirochaetaceae*	*Spirochaeta*	ROI2	G	↑
Cecum	*Actinobacteria*	*Microbacteriaceae*	*Microbacterium*	ROI2	F, G	↑
		*Coriobacteriaceae*	Uncultured	ROI2	F, G	↑
	*Bacteroidetes*	*Porphyromonadaceae*	*Paludibacter*	ROI2	G	↑
		RF16	Uncultured	ROI1, ROI2	F, G	↑
		*Rikenellaceae*		ROI2	F	↑
			dgA 11 gut group	ROI2	G	↑
			RC9 gut group	ROI2	G	↑
	*Cyanobacteria*	4c0d, uncultured	Uncultured	AT	P, F, G	↑
		*Gastranaerophilales*, uncultured	Uncultured	ROI2	F, G	↑
	*Firmicutes*			ROI1	P	↓
		*Erysipelotrichaceae*	Uncultured	ROI2	F, G	↑
			*Asteroleplasma*	ROI2	G	↑
		Family XIII	Uncultured	ROI2	G	↑
		*Lachnospiraceae*	*Butyrivibrio*	ROI2	G	↓
		*Peptococcaceae*	*Peptococcus*	ROI2	F, G	↑
		*Ruminococcaceae*		ROI2, AT	F	↑
			*Incertae sedis*	ROI2	G	↑
			*Oscillospira*	ROI1	G	↓
			*Papillibacter*	ROI2	G	↑
		vadinBB60	Uncultured	ROI2	G	↑
		*Streptococcaceae*	*Streptococcus*	ROI2	F, G	↓
		*Veillonellaceae*	Uncultured	ROI1	F, G	↓
	*Lentisphaerae*	RF12 gut group, uncultured	Uncultured	ROI2	F, G	↑
	*Proteobacteria*			ROI1	P	↑
		*Rhodospirillaceae*		ROI1	F	↑
		*Alcaligenaceae*	*Sutterella*	ROI1	F, G	↑
		*Oxalobacteraceae*	*Noviherbaspirillum*	ROI2	F, G	↑
		GR WP33 58	Uncultured	ROI2	F, G	↑
		*Helicobacteraceae*	*Helicobacter*	ROI2	F, G	↑
		*Succinivibrionaceae*	*Ruminobacter*	ROI1	F, G	↑
	*Spirochaetes*	*Spirochaetaceae*	*Treponema*	AT	G	↓
	*Synergistetes*	*Synergistaceae*	*Pyramidobacter*	ROI2	F, G	↑
	*Tenericutes*	*Anaeroplasmataceae*	*Anaeroplasma*	ROI1	F, G	↑
		NB1 n, uncultured	Uncultured	ROI2	F, G	↑
	*Verrucomicrobia*	*Puniceicoccaceae*	*Puniceicoccus*	ROI2	P, F, G	↑
		vadinHA64, uncultured	Uncultured	ROI2	F, G	↑
		*Verrucomicrobiaceae*	*Akkermansia*	ROI1	F, G	↑

a A total of 104 RFI-associated microbial composition differences were observed across the three geographic locations.

b All phylum-level differences between high- and low-RFI pigs are shown in Fig. S2 in the supplemental material.

c AT, Austria; ROI, Republic of Ireland (batches 1 and 2).

d Significantly different (*P* < 0.05) at the phylum (P), family (F), and/or genus (G) level.

e Arrows indicate relative-abundance differences in low-RFI pigs compared to high-RFI pigs at the same geographic location (↓, lower relative abundance; ↑, higher relative abundance).

### RFI-associated bacterial taxa in the fecal and intestinal microbiota of pigs ranked on RFI at different geographic locations.

**FIG 4:**
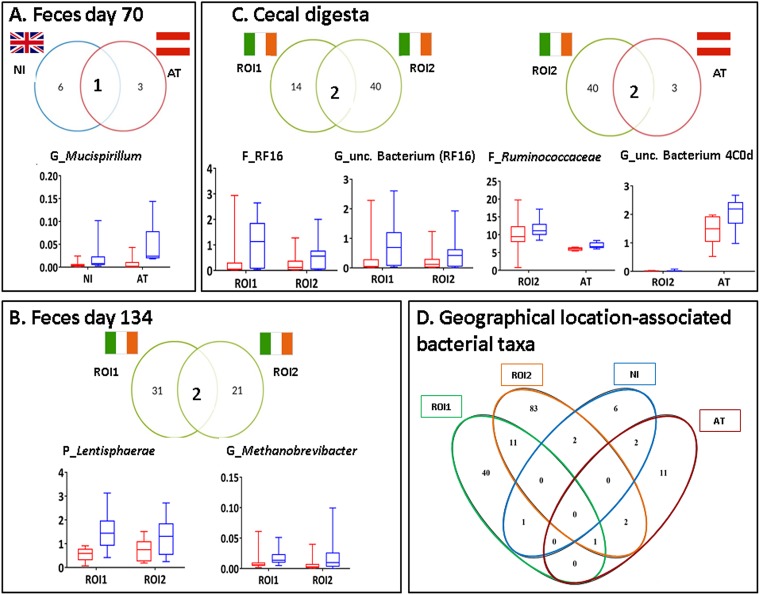
(A to C) RFI-associated bacterial taxa common to more than one geographic location in feces from pigs at day 70 (A) and day 134 (B) and in cecal digesta (C). Median relative abundances (percent) of these taxa in low-RFI (blue boxes) and high-RFI (red boxes) pigs are also shown. (D) RFI-associated bacterial taxa shared across geographic locations, irrespective of sample type. Only taxa that were significantly different between high- and low-RFI ranks across locations are depicted (*P* ≤ 0.05). Digesta was not sampled from NI pigs. P, phylum; F, family; G, genus.

Although none of the 188 RFI-associated taxonomic differences within the fecal and/or intestinal microbiota were common to all four groups of pigs (Fig. 4D), seven taxa were found to be enriched in low-RFI (more-feed-efficient) pigs at more than one geographic location or in the two different batches reared in ROI (*P* < 0.05) (Fig. 4). In the feces collected on day 70 of age, *Mucispirillum* (from *Deferribacteres*) was more abundant in low- than in high-RFI pigs from both NI (2-fold) and AT (15-fold) (*P* < 0.05). On day 134 of age, the phylum *Lentisphaerae* was enriched in the feces of low-RFI pigs in both ROI1 and ROI2 (ROI1, 2.4-fold; ROI2, 1.7-fold), as was the genus *Methanobrevibacter* (from *Euryarchaeota*) (ROI1, 2.3-fold; ROI2, 3.3-fold [*P* < 0.05]). In the cecal digesta, four cross-locational RFI-associated taxa were observed. In both ROI1 and ROI2, the bacterial family RF16 and an uncultured bacterium from this family were enriched in low-RFI pigs (22-fold for ROI1 and 5-fold for ROI2, and 14-fold for ROI and 4-fold for ROI2, respectively [*P* < 0.05]). The RF16 family was also 20 times more abundant in the feces of low-RFI pigs than in the feces of high-RFI pigs on day 134 of age in ROI1 (*P* < 0.05) (data not shown). In addition, two similar OTUs belonging to an uncultured bacterium from RF16 were found at a higher abundance (∼20-fold) in the feces on day 134 of age in low-RFI pigs from both ROI1 and AT than their high-RFI counterparts (*P* < 0.05). Furthermore, in the cecum, low-RFI pigs in ROI2 and AT had higher abundances of the family *Ruminococcaceae* (1.2-fold and 1.1-fold, respectively) and an uncultured bacterium from the *Cyanobacteria* (10-fold and 1.5-fold, respectively). However, the *Ruminococcaceae* family was the only cross-locational RFI-associated taxon with a median relative abundance of >5%. In contrast, in the ileum, no common RFI-associated differences were observed across geographic locations.

When data from the four rearing environments were combined, a number of bacterial genera with a relative abundance of >0.1% were exclusively found in low-RFI pigs at each time point (Fig. S3). In the feces of pigs at day 70 of age, there were eight low-RFI-specific genera; in the feces at slaughter, there were two; in the ileal digesta, there were nine; and in cecal digesta, there were seven genera exclusive to low-RFI pigs.

10.1128/mSystems.00324-18.3 FIG S3 Genera found exclusively in all low-residual-feed-intake (RFI)-ranked pigs across all geographic locations in feces at 70 days (A) and 134 days (B) of age and in ileal (C) and cecal (D) digesta. Data from all geographic locations were taken together. unc, uncultured; und, unidentified. Taxa present at a <0.1% relative abundance were not included in the analysis. Download FIG S3, DOCX file, 0.8 MB. Copyright © 2019 McCormack et al. 2019 McCormack et al. This content is distributed under the terms of the Creative Commons Attribution 4.0 International license.

### Predicted microbial pathways in the feces and digesta of pigs ranked on RFI reared at different geographic locations.

**FIG 5:**
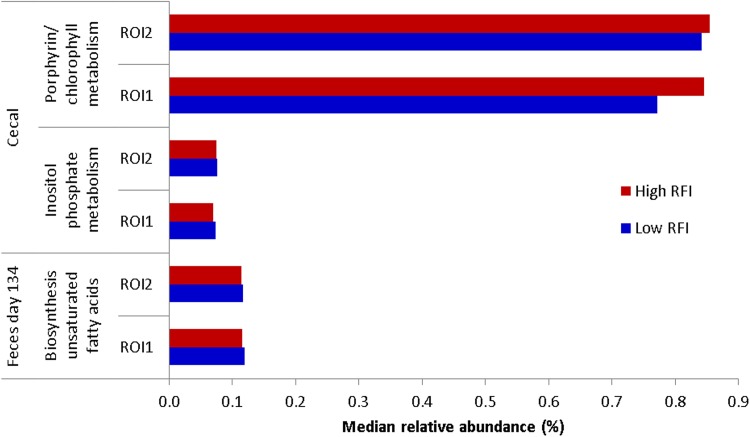
Common RFI-associated predicted microbial pathways in low- and high-RFI pigs across geographic locations. Only the predicted pathways that were significantly different between high- and low-RFI ranks across locations are depicted.

Potential functionality of the intestinal microbiota was inferred using the phylogenetic investigation of communities by reconstruction of unobserved states (PICRUSt) package. One hundred and two predicted microbial pathways differed significantly in relative abundance between high- and low-RFI pigs across geographic locations and by sample type and were subsequently grouped into major functional categories (Fig. S4). These pathways were present at a very low relative abundance (≤2.1%) and were mainly related to metabolism, especially carbohydrate metabolism in the feces at day 70 of age, energy metabolism in the feces of pigs at day 134 of age and in the cecum, and nucleotide metabolism in the ileum (Fig. S4). Depending on the age of the pig, there was also a relatively high representation of pathways related to genetic information processing (e.g., replication and repair or transcription). There were differences in the abundances of Kyoto Encyclopedia of Genes and Genomes (KEGG) orthology (KO) functions between high- and low-RFI pigs across locations. In the cecal and ileal digesta, for most of the locations, most pathways were at a higher relative abundance in the low-RFI pigs than in their high-RFI counterparts. In the feces of pigs at day 70 of age, most of the differentially abundant pathways were found in the pigs from NI, whereas for the rest of the time points, most of the differences were assigned to ROI1 and ROI2 pigs. However, none of the differentially abundant predicted microbial pathways were common to all geographic locations, and only three followed the same trend in both ROI batches: biosynthesis of fatty acids in the feces at day 134 of age and inositol phosphate metabolism and porphyrin/chlorophyll metabolism in the cecal digesta (*P* < 0.05) (Fig. 5). The latter was most abundant in the high-RFI (less-feed-efficient) pigs, while the abundances of the other two pathways were higher in the low-RFI pigs (*P* < 0.05). The inositol phosphate metabolism pathway, belonging to the core carbohydrate metabolism function, was also differentially abundant in both the ileal and cecal digesta of pigs from both ROI batches.

10.1128/mSystems.00324-18.4 FIG S4 Predicted functionality of intestinal microbiota for high- and low-RFI pigs across geographic locations and by sample type (feces [A] and cecal digesta [B]). Pathways shown are those found to be differentially abundant between high- and low-RFI pigs (*P* < 0.05). ROI, Republic of Ireland; NI, Northern Ireland; AT, Austria. Pathways were grouped into major functional categories as follows: M1, carbohydrate metabolism; M2, metabolism of cofactors and vitamins; M3, energy metabolism; M4, lipid metabolism; M5, amino acid metabolism; M6, glycan biosynthesis and metabolism; M7, biosynthesis of other secondary metabolites; M8, metabolism of terpenoids and polyketides; M9, xenobiotic biodegradation and metabolism; M10, metabolism of other amino acids; M11, nucleotide metabolism; E, environmental information processing; G, genetic information processing; C, cell motility. Download FIG S4, DOCX file, 0.1 MB. Copyright © 2019 McCormack et al. 2019 McCormack et al. This content is distributed under the terms of the Creative Commons Attribution 4.0 International license.

### Volatile fatty acid concentrations in pigs ranked on RFI reared at different geographic locations.

**FIG 6:**
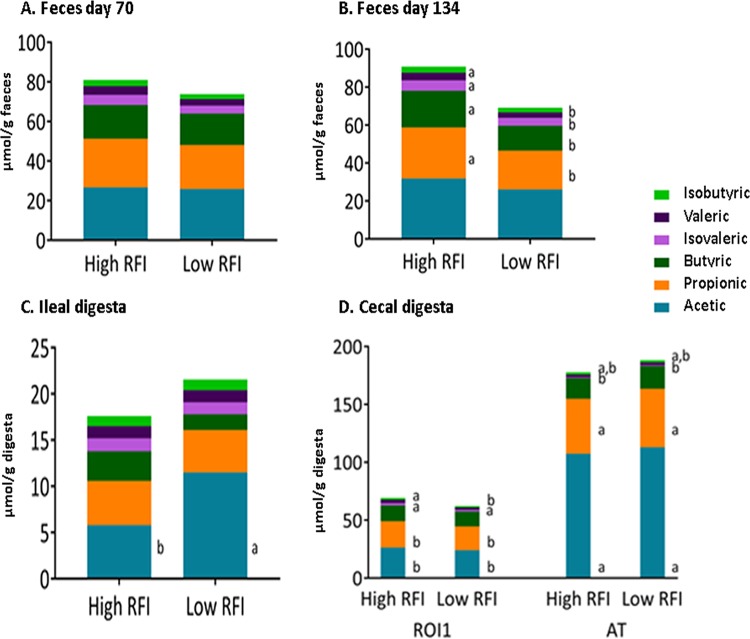
Effect of ranking pigs on RFI on volatile fatty acid (VFA) concentrations (millimoles per gram) in feces of ROI1 pigs at day 70 (A) and day 134 (B) of age and in ileal digesta (C) and cecal digesta (D) of ROI1 and AT pigs. VFAs that do not share a superscript letter (a and b) are significantly different from each other (*P* < 0.05).

Concentrations of volatile fatty acids (VFAs) were determined in feces collected from pigs on days 70 and 134 of age, in the ileal and cecal digesta from pigs in ROI1, as well as in the cecal digesta from pigs in AT (Fig. 6). No differences were observed between RFI ranks in feces collected from pigs on day 70 of age. However, low-RFI pigs had lower concentrations of total VFAs as well as butyric and propionic acids (*P* < 0.05) and a tendency toward lower valeric acid (*P* = 0.07) and isovaleric acid (*P* = 0.09) concentrations in the feces collected at day 134 of age. In the ileal digesta, low-RFI pigs had higher concentrations of total VFAs and acetic acid (*P* < 0.05). In the cecum, a strong influence of geographic location was observed for all VFAs measured, apart from butyric and isobutyric acids. Pigs in AT had higher concentrations of total VFAs, as well as higher acetic and propionic acid concentrations, compared to ROI1 pigs (*P* < 0.05). The pigs in AT had lower concentrations of isovaleric acid than did pigs from ROI1 (*P* < 0.05). The concentration of valeric acid was lower in low-RFI pigs from ROI1 than in their high-RFI counterparts and pigs from AT (*P* < 0.05).

### Bacterial taxa correlated with RFI values and volatile fatty acid concentrations in pigs ranked on RFI reared at different geographic locations.

A correlation analysis was performed between the intestinal microbiota composition, at the phylum and genus levels, and RFI values and VFAs that differed significantly between RFI ranks. Those correlations that were significant are shown as a heat map in Fig. S5. None of the phyla or genera that were RFI associated in this study were significantly correlated with RFI value. At the phylum level, only the phylum *Verrucomicrobia* in the cecum was negatively correlated with a low RFI value (*R* = −0.56). At the genus level, *Megasphaera* (*R* = −0.57) and an uncultured genus from the *Rhodospirillaceae* (*R* = −0.54) in the ileum were correlated with a low RFI value.

10.1128/mSystems.00324-18.5 FIG S5 Heat map showing Spearman correlations between bacterial phyla and genera and physiological parameters measured. * indicates significance at a *P* value of ≤0.05. Red indicates a negative correlation, and blue indicates a positive correlation. ^1^Volatile fatty acid concentrations were measured in Republic of Ireland batch 1 (ROI1) (feces at day 134 and ileal and cecal digesta) and Austria (AT) (cecal digesta) pigs only. ^2^Residual feed intake (RFI), calculated between days 70 and 120 of age for all pigs. Download FIG S5, DOCX file, 0.6 MB. Copyright © 2019 McCormack et al. 2019 McCormack et al. This content is distributed under the terms of the Creative Commons Attribution 4.0 International license.

In relation to correlations between microbes and VFA concentrations, butyric acid in the feces collected from ROI1 pigs at day 134 of age was positively correlated with *Mycoplasma* (*R* = 0.69) but negatively correlated with *Pyramidobacter* (*R* = −0.59). Valeric acid was negatively correlated with an uncultured genus from *Verrucomicrobia* subdivision 5b (*R* = −0.70), but isovaleric acid was positively correlated with *Collinsella* (*R* = 0.67). In the ileum of ROI1 pigs, there was a negative correlation between acetic acid and *Clostridium sensu stricto* 1 (*R* = −0.70).

### Salivary cortisol concentrations and hematological and biochemical parameters in pigs ranked on RFI reared at different geographic locations.

The salivary cortisol concentration, measured only in pigs from ROI1 just prior to slaughter (day 130 of age), tended to be lower in low-RFI (more-feed-efficient) pigs (*P* = 0.06) (Table 1). In pigs from ROI1, ROI2, and AT, no significant differences were observed according to RFI rank for serum biochemistry measures or for any of the hematological parameters measured (*P* > 0.05) (Table S1), and most values were within the normal ranges previously reported in growing pigs (24).

10.1128/mSystems.00324-18.6 TABLE S1 Hematological and serum biochemical^1^ parameters in pigs ranked on residual feed intake (RFI) in ROI^2^ and AT^3^ pigs. Download Table S1, DOCX file, 0.02 MB. Copyright © 2019 McCormack et al. 2019 McCormack et al. This content is distributed under the terms of the Creative Commons Attribution 4.0 International license.

### Ileal immunological capacity and serum haptoglobin and cecal lipopolysaccharide levels in pigs ranked on RFI reared at different geographic locations.

No significant differences were observed between high- and low-RFI pigs from ROI1 for any of the ileal lymphocyte populations or cytokine concentrations measured from lamina propria lymphocytes (LPL) and intraepithelial lymphocytes (IEL), either control or mitogen stimulated (Table S2). Most of the lymphocyte populations in pigs from both RFI ranks were within the ranges previously reported for younger, healthy/control pigs (25, 26). Similarly, no significant differences were observed between RFI ranks across geographic locations for serum haptoglobin or cecal lipopolysaccharide (LPS) concentrations (*P* > 0.05) (Table S3).

10.1128/mSystems.00324-18.7 TABLE S2 Pooled ileal intraepithelial lymphocyte (IEL) and lamina propria lymphocyte (LPL) populations (percent),^1^ with or without mitogen stimulation, from ROI1 pigs ranked based on residual feed intake (RFI) and cytokine production (picograms per milliliter) from these cells. Download Table S2, DOCX file, 0.01 MB. Copyright © 2019 McCormack et al. 2019 McCormack et al. This content is distributed under the terms of the Creative Commons Attribution 4.0 International license.

10.1128/mSystems.00324-18.8 TABLE S3 Cecal lipopolysaccharide^1^ and serum haptoglobin^2^ concentrations in pigs ranked on residual feed intake (RFI) across geographic locations. Download Table S3, DOCX file, 0.01 MB. Copyright © 2019 McCormack et al. 2019 McCormack et al. This content is distributed under the terms of the Creative Commons Attribution 4.0 International license.

## DISCUSSION

Recently, a number of studies have demonstrated an association between FE and intestinal microbiota composition and function in pigs (12, 13, 16, 19, 21). However, very few of the FE-associated microbial taxa identified are common across studies. This is most likely due to the differences in diet, genetics, management strategies, and FE metrics used across rearing environments. Following on from this, the intestinal microbial profile of pigs ranked on RFI from three geographic locations was assessed in the present study but with these factors controlled in an attempt to find reliable cross-locational microbial biomarkers for FE in pigs. However, across geographic locations, few potential microbial biomarkers that were common to more than one site were identified, and most of these were different from those pinpointed in our previous study (12). The fact that ranking based on RFI was performed during different stages of the production cycle, i.e., days 70 to 120 of age in the present study compared to between weaning (∼28 days old) and day 154 of age in the previous study, could help to explain the discrepancies. Moreover, in the present study, none of the RFI-associated differences in intestinal microbiota composition found were common across all geographic locations. Similarly, studies in chickens have highlighted the challenge of finding common FE-associated microbes across locations (27) and even across batches from within the same location (28).

The limited cross-locational RFI-associated differences in the intestinal microbiota were perhaps due to variation in the core microbiome in pigs at each location, as illustrated by the location-specific diversity found in the PCoA plots. Similarly, Metzler-Zebeli et al. found that geographic location had more of an influence on intestinal size, structure, and functionality than RFI in the same pigs (29). It has previously been shown that poultry raised in different environments have different microbiota (28). However, to date, this has not been shown for pigs, although it is likely to occur considering that even the pen in which a pig is housed impacts the intestinal microbiota (13). This also seems to be the case in the present study, despite the fact that external factors, including diet specification, diet phases, genetics, and management protocols, were the same. It is also well known that the microbiome of the sow influences the microbiota composition of her progeny (30). In the present study, we attempted to account for this when looking at RFI-associated microbiota differences by selecting pigs of both high and low RFI from within the same litter. Nonetheless, litter origin is also likely to have contributed to the microbiota differences observed across geographic locations. In addition, it is likely that the housing environment and health status differences (31), however subtle, between locations also played a part in the differences observed. Overall, these maternal and environmental influences, as well as the interindividual variability in intestinal microbiota highlighted by Yang et al. (13), make it difficult to find reliable universal biomarkers for FE.

The overall intestinal microbiota composition showed a high abundance of *Firmicutes* and *Bacteroidetes*, which mirrors the fact that these are commonly identified as major constituents of the core pig microbiome (20). The lower relative abundance of the phylum TM7 and its genus “*Candidatus* Saccharimonas” observed in the low-RFI pigs from ROI2 at day 134 resembles previous findings from our group (12). This phylum is diverse, comprising ubiquitous members with potential proinflammatory activity previously found to be enriched in humans with inflammatory bowel disease (32).

Although none of the RFI-associated differences in microbiota composition were common to all geographic locations, seven taxa were found to differ in abundance between high- and low-RFI pigs at more than one geographic location or from the two different batches reared at the same location in ROI. However, within RFI rank, variation between pigs was observed; these taxa differences appear to explain, at least in part, differences in FE and could potentially be used as microbial biomarkers for FE. For example, the low-RFI-associated microbial taxa have a major role in core metabolism of carbohydrates. In feces collected from pigs at day 70 of age, the abundance of *Mucispirillum*, which is a mucin degrader, was found to be higher in low-RFI pigs from NI and AT. However, too much of a shift toward this genus could be harmful, causing disruption of the mucus layer (33). In feces collected from pigs at day 134 of age, *Methanobrevibacter* was more abundant in the highly efficient than in the poorly efficient pigs in both ROI batches. This genus has previously been positively correlated with fiber digestibility in pigs (34) and also plays an important role in methane production (35). Recently, it was found to be enriched in healthy humans compared to those with irritable bowel syndrome (36), and a link with leaner phenotypes in humans is also noteworthy (37). The phylum *Lentisphaerae*, enriched in low-RFI pigs, has previously been associated with improved health in humans, which might suggest a healthier gut microbiome here (38). This phylum has also been associated with improved FE and, specifically, weight gain, albeit in cattle (39), which would suggest a benefit in further investigating its role in improving FE in pigs. In the cecal digesta, the family *Ruminococcaceae*, which has a central role in the fermentation of carbohydrates, including cellulose (40, 41), and in the production of butyrate, was identified as an RFI-associated taxon in pigs from both AT and ROI2. Moreover, this family represents a core taxon with a relative abundance of 5 to 10%, and it has previously been linked with improved FE in pigs (13, 19). Finally, an uncultured bacterium from the RF16 family along with the RF16 family itself (from *Bacteroidetes*) were present at higher relative abundances in low-RFI pigs and exclusively found in low-RFI pigs. Although there is little information regarding the role of OTUs from this family within the intestinal community, they were enriched in pigs fed a diet high in complex carbohydrates (42), which could suggest an enriched biofunction of carbohydrate catabolism. In work previously conducted by this group, species from *Bifidobacterium* found exclusively in low-RFI pigs (12) were again found exclusively in low-RFI pigs in the present study. Additionally, two members of the *Clostridiales*, an uncultured member of the vadinBB60 family and the genus *Cellulosilyticum*, were found to be present only in low-RFI pigs here, and this was also found in previous work by our group (12). Moreover, *Bacteroides* in the feces at slaughter may be a potential microbial marker for RFI, as low-RFI pigs had a high relative abundance compared to high-RFI pigs in both this and our previous study (12).

The predicted RFI-associated microbial function data agreed with the differential compositional data, showing that most predicted pathways enriched in low-RFI pigs were related to core metabolism, including carbohydrate, energy, and nucleotide metabolism. Perhaps not surprisingly, this is in agreement with the findings of other recent studies on FE-associated microbiota in pigs (13, 19). The findings from a hepatic gene expression study with FE-divergent pigs, suggesting that carbohydrate biofunction is enriched in highly-feed-efficient pigs (7), also complement the microbial functionality found here, with the phosphorylation of inositol pathway being FE associated in both studies.

Microbial metabolites such as VFAs have a key role in modulation of host cells and also constitute an extra source of energy in the hindgut of the host. The concentration of VFAs present impacts the host phenotype, with, for example, higher concentrations of VFAs in the feces associated with obesity (43). The fact that fecal VFA concentrations were lower in low-RFI pigs could suggest increased colonic absorption, indicating better utilization of bacterial fermentation end products in the large intestine (44). However, the contribution of colonic VFAs to energy supply in young pigs, as was the case here, is low relative to that in adult pigs (e.g., sows or breeding boars). There appeared to be a strong influence of rearing environment on the cecal VFA concentrations measured in the present study, with ROI1 pigs having lower concentrations of most VFAs than AT pigs.

Apart from intestinal microbiota profiles and associated microbial metabolites, the level of salivary cortisol was the only other measure found to be RFI associated (lower in low-RFI pigs). This suggests that cortisol could be useful as a biomarker for improved FE in pigs, as has been previously suggested for cattle (3). Despite the absence of a link between ileal immune competence and FE in our study, an adequate number of ileal immune cells (i.e., within normal ranges previously found for healthy, control pigs [25, 26]) indicates an ability to fight off disease while maintaining optimum growth performance. Likewise, no RFI-associated differences were found for cecal concentrations of the bacterial endotoxin LPS, a potent immunogen, high serum concentrations of which have previously been linked to poor FE (45). Serum concentrations of the acute-phase protein haptoglobin also did not differ between RFI ranks in the present study, although they were previously found to be lower in low-RFI pigs (46). In addition, while previous work has shown that low-RFI pigs were more efficient in terms of producing lower but sufficient levels of blood lymphocytes, monocytes, and white blood cells than high-RFI pigs (5), no RFI-associated hematological differences were found in the present study.

### Conclusions.

In conclusion, the FE-associated bacterial taxa consistently found across rearing environments may have a role to play in improving FE in pigs, mainly because of their importance in relation to carbohydrate metabolism. In addition, methanogenic members of the *Archaea* (*Methanobrevibacter*) are also likely to shape FE in pigs. In the future, these FE-associated taxa could potentially be used as probiotics or targeted by dietary means as a strategy for improving FE in pigs. Alternatively, they could be exploited as potential predictive biomarkers for porcine FE. However, the unculturable nature of some of these taxa together with location-specific findings highlight the challenges associated with the translation of our data into a set of reliable usable biomarkers and/or potential probiotics for pigs. Furthermore, the complex interplay between microbes within the gut ecosystem, in particular in the cecum, makes the cause-effect relationship intricate. Therefore, the microbial taxa identified in the present study cannot be interpreted as definitive determinants for FE in pigs, without additional studies. Moreover, consortia of bacterial species, rather than individual taxa, may be more likely to predict FE.

## MATERIALS AND METHODS

### Ethical approval.

The trials in ROI were approved by the animal ethics committees of Teagasc (TAEC9/2013) and Waterford Institute of Technology (13/CLS/02), and an experimental license (number AE1932/P004) was obtained from the Irish Health Products Regulatory Authority (HPRA). The trial in NI was conducted under project licenses PPL 2751 and PPL 2781, obtained from the Department of Health, Social Services and Public Safety (DHSSPS), which adhered to the Animals (Scientific Procedures) Act of 1986. The trial in AT was approved by the institutional ethics committee and the national authority according to paragraph 26 of the Law for Animal Experiments, Tierversuchsgesetz 2012 (TVG 2012) (GZ 68.205/0058-WF/II/3b/2014). All animal procedures were performed according to European Union Directive 2010/63/EU on the protection of animals used for scientific purposes (47).

### Animal management, performance records, and sampling.

**FIG 7:**
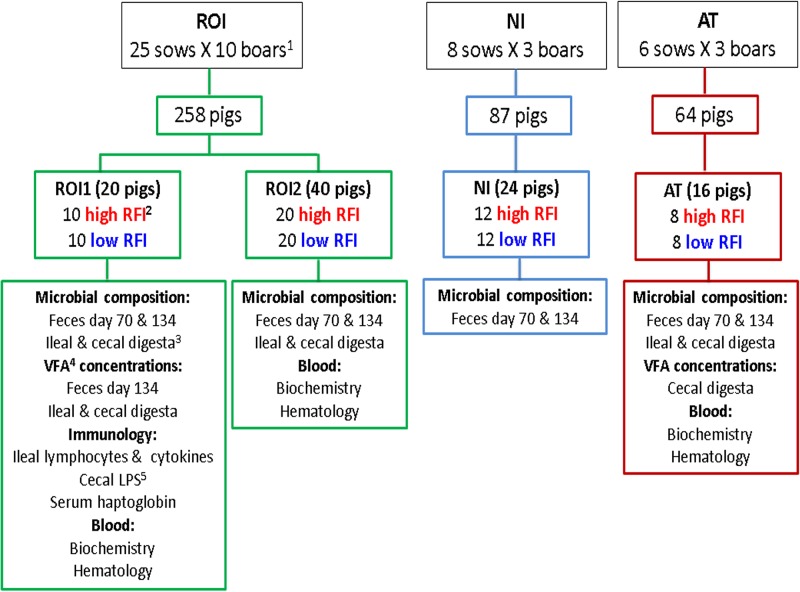
Schematic showing pig selection based on RFI and sampling procedure across geographic locations. ^1^one common boar was used across the three locations to minimize genetic variation; ^2^pigs were ranked on RFI at between 70 and 120 days of age; ^3^pigs were slaughtered at ∼134 days of age, and ileal and cecal digesta were collected; ^4^volatile fatty acids; ^5^lipopolysaccharide.

A schematic illustration depicting animal management, selection, and sample collection is shown in Fig. 7. Four trials were conducted across three geographic locations: ROI, NI, and AT. A total of 39 sows (Large White × Landrace), across all three locations (25 in ROI, 8 in NI, and 6 in AT), were blocked by body weight and inseminated with semen from individual boars (Maxgro; Hermitage Genetics Co., Kilkenny, Ireland). One common boar was used across the three locations, with an additional 10 boars specific to ROI, 3 specific to NI, and another 3 specific to AT.

Subsequent offspring comprised 369 piglets: 218 in ROI (two batches, ROI1 [*n* = 80] and ROI2 [*n* = 138]), 87 in NI, and 64 in AT. All pigs were weaned at 28 ± 3 days of age, housed in groups of intact litters, and fed via feed intake recording equipment (FIRE) feeders (Schauer Agrotronic, Wels, Austria) (ROI1, 8 feeders with 7 to 10 pigs/pen; ROI2, 12 feeders with 10 to 13 pigs/pen; NI, 8 feeders with 10 to 12 pigs/pen; AT, 6 feeders with 9 to 12 pigs/pen). Pigs were fed the same sequence of diets (starter, link, weaner, and finisher), with the same diet specifications across all three geographic locations. Water and feed were provided on an *ad libitum* basis. The ingredients and chemical compositions of all experimental diets are shown in Table S4 in the supplemental material.

10.1128/mSystems.00324-18.9 TABLE S4 Composition and chemical analysis of diets used in the study (as-fed basis) (in grams per kilogram). Download Table S4, DOCX file, 0.01 MB. Copyright © 2019 McCormack et al. 2019 McCormack et al. This content is distributed under the terms of the Creative Commons Attribution 4.0 International license.

Individual body weight was recorded every week, and voluntary feed intake was recorded daily between day 70 and day 120 of age, to calculate growth performance: ADFI, ADG, and FCE. Ultrasound measurements of back fat (BF) depth and muscle depth (MD) were taken at the 3rd and 4th last rib every week, using a Piglog 105 instrument (Carometec, Herlev, Denmark) for ROI, a Sonoscope A5 instrument (Keebomed, Mount Prospect, IL) for NI, and a Renco lean meater (Renco Corporation, Minneapolis, MN) for AT. All pigs were checked at least twice daily; any pigs showing signs of illness were treated as appropriate, and the details were recorded.

After day 120 of age, RFI was calculated for each pig (between days 70 and 120 of age), and extremes for RFI were selected within litter and gender. The RFI is a metric of FE that assesses the difference between the actual and predicted feed intake, with low-RFI animals being the most feed efficient. At each geographic location, RFI was calculated as the residuals from a least-squares regression model of ADFI on ADG, metabolic live weight (body weight^0.75^), gender, and all relevant two-way interactions as well as the effects of BF depth and MD. Pigs were ranked as having either high or low RFI, with a minimum spread of 2 standard deviations from the mean (within location and batch) between RFI ranks. A total of 100 pigs (60 from ROI [ROI1, 20; ROI2, 40], 24 from NI, and 16 from AT) were selected for sampling. A schematic depicting the selection of pigs is shown in Fig. 7.

Individual fecal samples were collected from all selected pigs following rectal stimulation on days 70 and 120 of age, immediately snap-frozen in liquid nitrogen, and stored at −80°C for subsequent microbiota and VFA analyses. Two weeks after selection of RFI extremes (approximately day 134 of age), pigs were slaughtered by CO_2_ stunning followed by exsanguination. Hot-carcass weight was recorded immediately following slaughter and was multiplied by 0.98 to obtain cold-carcass weight. The kill-out percentage was calculated as (cold carcass weight/body weight at slaughter) × 100. Back fat depth and MD at slaughter were measured 6 cm from the edge of the split back at the 3rd and 4th last ribs using a Hennessy grading probe (Hennessy and Chong, Auckland, New Zealand). Lean-meat yield was estimated according to the following formula: lean meat yield = 60.30 − 0.847 *X*_1_ + 0.147 *X*_2_ (where *X*_1_ is back fat depth [millimeters] and *X*_2_ is muscle depth [millimeters]). Digesta samples were collected from the terminal ileum (15 cm proximal to the ileocecal junction) and the cecum (terminal tip) from the selected pigs in ROI and AT. Samples were immediately snap-frozen in liquid nitrogen and stored at −80°C for subsequent microbiota and VFA analyses.

### Salivary cortisol analysis.

On day 130 of age, saliva samples were collected from ROI1 pigs by allowing them to chew on a cotton bud (Salivette; Sarstedt Co., Wexford, Ireland). Cortisol concentrations were determined in duplicate using a high-sensitivity enzyme-linked immunosorbent assay (ELISA) kit (Salimetrics Europe Ltd., Suffolk, UK) according to the manufacturer’s instructions.

### Hematology and serum biochemistry analyses.

Blood was collected from ROI and AT pigs during exsanguination at the slaughter plants for hematology and biochemistry analyses. For hematological analysis, blood was collected in vacuette tubes (from Labstock, Dublin, Ireland, for ROI and from Sarstedt, Nürnbrecht, Germany, for AT) containing EDTA to prevent clotting and analyzed within 4 h using a Beckman Coulter Ac T Diff analyzer (Beckman Coulter Ltd., High Wycombe, UK) for ROI pigs and a ProCyte dx hematology analyzer (Idexx Laboratories, Inc., Westbrook, ME, USA) for AT pigs.

For biochemical analysis, blood was collected from ROI pigs in vacuette tubes (Labstock) and allowed to clot at room temperature prior to centrifugation at 1,500 × *g* for 10 min. The serum was then collected and stored at −80°C for subsequent analysis. Serum samples were analyzed using an ABS Pentra 400 clinical chemistry analyzer (Horiba, ABX, North Hampton, UK) for total protein, blood urea nitrogen, cholesterol, glucose, triglycerides, creatinine, and creatine kinase. The analyzer was calibrated according to the manufacturer’s instructions, and every fifth sample was run in duplicate to determine analyzer accuracy. For AT pigs, blood was collected in serum collection tubes (Sarstedt, Nürnbrecht, Germany) and centrifuged at 1,811 × *g* for 10 min. Serum was then collected and stored at −80°C for subsequent analysis of levels of blood urea nitrogen, glucose, triglycerides, and cholesterol, which were determined by standard enzymatic colorimetric analysis using a clinical chemistry autoanalyzer, as outlined previously (48).

### Immunological analyses.

Ileal tissue (2 to 3 cm) was collected 15 cm proximal to the ileocecal junction from ROI1 pigs at slaughter, placed in Hanks’ balanced salt solution (HBSS; Sigma-Aldrich Co., Wicklow, Ireland), and put on ice. The LPL and IEL were isolated from the ileal tissue as previously described (25) and pooled. Briefly, cells were isolated from the mucosa and submucosa of the porcine ileal tissue samples, suspended at 10^6^ cells/ml in complete medium (Iscove’s modified Dulbecco’s medium [IMDM] plus GlutaMAX [Invitrogen], 20% heat-inactivated fetal bovine serum [FBS], 100 U/ml penicillin, and 100 mg/ml streptomycin), and transferred into 24-well plates (Sarstedt, Nürnbrecht, Germany) in quadruplicate. Cells were then pooled, treated with phosphate-buffered saline (PBS) (control) or stimulated with phorbol myristate acetate (PMA) (25 ng/ml; Sigma-Aldrich) plus ionomycin (I) (1 μg/ml; Sigma-Aldrich) (PMA+I) (mitogen stimulated) at 37°C in a 5% (vol/vol) CO_2_-humidified atmosphere for 4 to 5 h, and incubated for 18 h. Cells were centrifuged at 1,230 × *g* for 20 min, and supernatants were stored at −80°C until cytokine analysis as outlined below.

Immunophenotyping was then performed on the pooled LPL and IEL that were washed and resuspended in 2% FBS–PBS using a BD FACSCanto II flow cytometer (BD Biosciences, Devon, UK), with at least 50,000 events acquired and analyzed. Data were analyzed using FACSDiva software (BD Biosciences). Primary and secondary antibodies were added at concentrations determined by previous titration, and incubations were performed in the dark at room temperature for 15 min. Antibodies used were CD45 fluorescein isothiocyanate (FITC) (lymphocyte marker) (AbD Serotec/Bio-Rad, Kidlington, UK) to verify the white blood cell population identified by light scatter, anti-porcine B cell marker phycoerythrin (PE) (Abcam, Cambridge, UK), CD3 phycoerythrin/cyanine 5 (Cy5) (T cell marker) (Abcam), anti-porcine CD14 PE/Cy7 (monocyte marker) (Abcam), mouse anti-porcine CD4a FITC (BD Biosciences), mouse anti-porcine CD8a (BD Biosciences), purified rat anti-pig γδ T lymphocytes (BD Biosciences), and goat anti-rat FITC (AbD Serotec/Bio-Rad). Proportions of B cells, total T cells, and monocytes were calculated as percentages of the total white blood cells that were identified by light scatter and verified with CD45 antibody to be 63.51% ± 24.49% positive in high-RFI pigs and 51.53% ± 16.45% positive in low-RFI pigs. The T cell subsets were calculated based on the percentage of CD3-positive cells.

Concentrations of interleukin-4 (IL-4), IL-6, IL-8, and tumor necrosis factor alpha (TNF-α) were subsequently determined in the supernatants from pelleted immune cells treated with PBS and PMA+I using a multiplex ELISA (R&D Systems, Minneapolis, MN, USA) in triplicate according to the manufacturer’s instructions.

### Haptoglobin in serum.

The concentration of haptoglobin was determined in serum samples collected from ROI1, AT, and NI pigs using a porcine-specific commercial ELISA kit (GenWay, San Diego, CA, USA) according to the manufacturer’s instructions. Serum samples were diluted 7,000- to 12,500-fold, depending on the actual haptoglobin concentrations in samples. Haptoglobin concentrations were determined in duplicate, and the intra-assay coefficient of variation was below 10%.

### Lipopolysaccharides in cecal digesta.

Concentrations of cell-free LPS in cecal digesta collected from all pigs were determined using the pyrochrome *Limulus* amebocyte lysate (LAL) assay (Associates of Cape Cod, Inc., East Falmouth, MA, USA) as previously described (49). After dilution and deproteinization by heating, the supernatants were used in the assay. Changes in the optical density of samples at 405 nm were measured against calibration curves using Pyros EQS software (Associates of Cape Cod, Inc.) after the addition of pyrochrome LAL reagent and incubation at 37°C. Reactions were run in duplicate, and the intra-assay coefficient of variation was <10%.

### Microbiota profiling.

Total DNA was extracted from fecal, ileal, and cecal samples using the QIAamp DNA stool minikit (Qiagen, Crawley, United Kingdom) according to the manufacturer’s instructions, apart from adding a beat beating step and increasing the lysis temperature to 95°C, to increase DNA yield (14).

Microbial profiling was performed using high-throughput sequencing of the V3-V4 region of the 16S rRNA gene (paired-end reads of 2 by 250 bp) on an Illumina MiSeq platform. The Illumina-recommended 16S metagenomic library preparation (Nextera) protocol was followed, except that the PCR mix volume was doubled in the first PCR step and the number of amplification cycles was increased to 30 instead of 25. Any samples with fewer than 40,000 post-quality reads on the MiSeq platform were removed from the analysis. Raw sequences were merged using Flash (with a minimum overlap of 30 bp and a minimum read length of 460 bp) and quality checked using the split libraries script (with default parameters) from the QIIME package, version 1.9.1. Reads with 97% sequence homology were clustered into OTUs by *de novo* OTU picking, and chimeras were removed with the 64-bit version of USEARCH (50). Subsequently, OTUs were aligned to the SILVA rRNA-specific database (version 111), and a phylogenetic tree was generated within QIIME. Alpha and beta diversity analyses were also performed using QIIME. Principal-coordinate analysis plots based on unweighted UniFrac distances were visualized using EMPeror v0.9.3-dev. Further downstream images were generated using the R package Phyloseq.

### Microbial function prediction.

The predicted functionality of the microbiota for each sample based on 16S rRNA data was determined using PICRUSt according to RFI rank, geographic location, and sample type. PICRUSt is a software tool which uses the 16S rRNA gene sequence to predict the functionality of microorganisms (51). Prediction of functions was inferred based on KEGG (52) and Clusters of Orthologous Groups of Proteins (COG) annotations, which assign annotations according to the database. The KO functions that were not bacterium related or for which the relative abundance was <0.01% for all of the RFI ranks were dismissed.

### Volatile fatty acid concentrations in feces and digesta.

Concentrations of VFAs (acetic, butyric, isobutyric, propionic, valeric, and isovaleric acids) were measured in triplicate in feces collected from pigs on days 70 and 134 of age and in ileal and cecal digesta from the ROI1 pigs, and in cecal digesta from AT pigs using gas chromatography (GC) (Agilent 5890 gas chromatograph for ROI1 and Fisons gas chromatograph model 8060 MS DPFC for AT). For samples analyzed in ROI, ∼8 g of sample was weighed, diluted with 5% trichloroacetic acid (TCA) (2.5× the weight of the sample), and centrifuged at 1,800 × *g* at 4°C for 10 min. One and a half milliliters of the resultant supernatant and 1.5 ml of the internal standard (0.043 M 3-methylvaleric acid in 0.15 M oxalic acid; Sigma-Aldrich) were mixed gently, and the mixture was filtered through a 0.45-μm filter (VWR International Ltd., Dublin, Ireland) into a labeled 8-mm amber GC vial (Antech Solutions Ltd., Waterford, Ireland) and stored at −80°C until analyzed, as previously described (12, 53).

For cecal digesta analyzed in AT, aliquots of 1 g were thawed on ice and mixed with 0.2 ml metaphosphoric acid (4.3 M; Sigma-Aldrich), 1 ml of double-distilled water, and 200 μl of the internal standard (0.024 M 4-methylvaleric acid in 4.3 M phosphoric acid; Sigma-Aldrich), and the mixture was centrifuged at 3,148 × *g* for 10 min. The clear supernatant was filtered through a 0.45-μm filter (VWR International Ltd.) into a labeled 8-mm GC vial and analyzed as previously described (54).

### Statistical analyses.

Growth performance parameters (weight, ADG, ADFI, and FCE) were analyzed using a mixed linear model in SAS 9.4. Fixed effects included in the model were RFI rank, geographic location, gender, and time period as well as their interactions. While adjusting for gender, sow was included as a random effect, and a repeated-measures model was used to describe correlations between time periods (weekly weight gain and feed intake recordings). Physiological parameters measured at only one time point (i.e., ileal lymphocyte populations, cytokine data, salivary cortisol concentrations, biochemical and hematological parameters, and haptoglobin and VFA concentrations) were also analyzed using a mixed linear model; the above-mentioned fixed effects were included in the model. Comparisons of means were undertaken using a Tukey correction for multiple testing. Residual diagnostics were made to ensure that the assumptions of the analysis were met.

Statistical differences for microbiota abundance at the phylum, family, and genus levels were calculated in R using the SILVA 16S-specific database (version 111) and estimated using the Kruskal-Wallis test for independent samples and the Wilcoxon rank test for paired samples. Corrections for multiple comparisons were made using the Benjamini-Hochberg method (55). Each geographic location was statistically analyzed separately.

The RFI values and VFA concentrations found to differ between low- and high-RFI pigs were correlated with the taxonomic relative abundance at the phylum and genus levels (at all time points for each geographic location where appropriate). Spearman correlation values were calculated in SAS, using the PROC CORR procedure, and *P* values were adjusted using the stepdown Bonferroni test.

For all statistical analyses conducted, significance was set at a *P* value of ≤0.05.

### Data availability.

The raw 16S rRNA gene sequence data generated from this study are available in the European Nucleotide Archive under accession number PRJEB22209.
