# Supplementary figures and images for: Porcine Feed Efficiency-Associated Intestinal Microbiota and Physiological Traits: Finding Consistent Cross-Locational Biomarkers for Residual Feed Intake
Source: mSystems. 2019 Jun 18;4(4):e00324-18. doi: 10.1128/mSystems.00324-18 (PMC6581691; doi:10.1128/mSystems.00324-18)

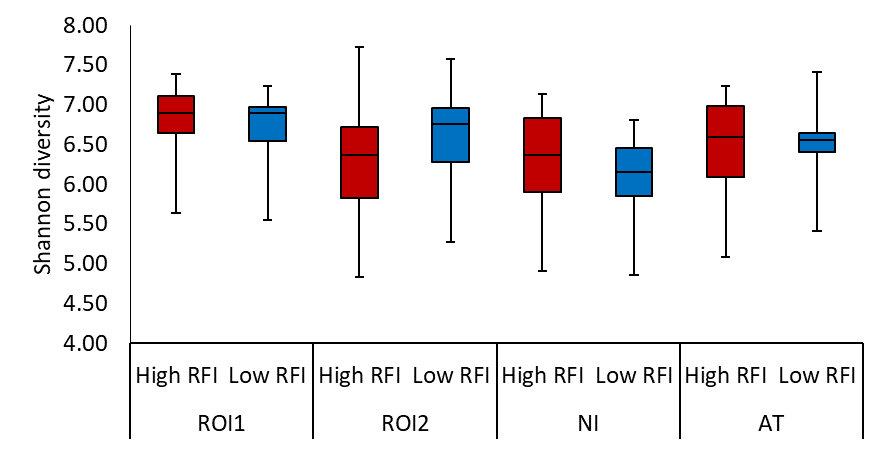


A. Feces at day 70

B. Feces at day 134


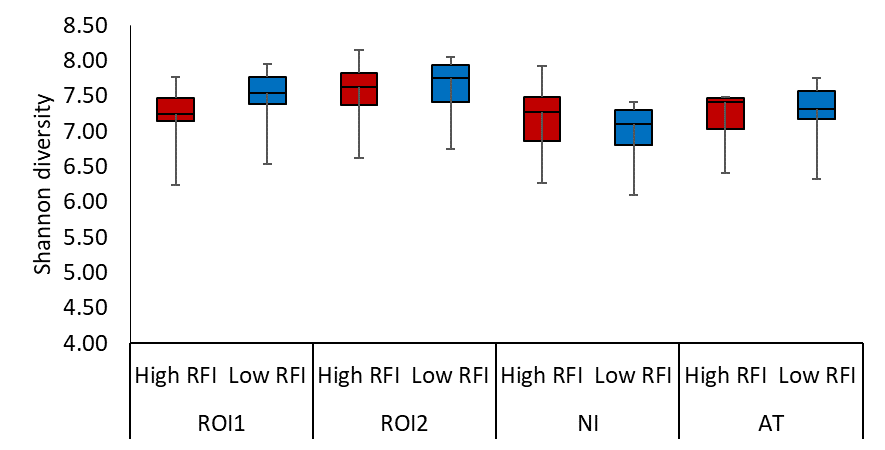

Supplement: FIG S1 [file mSystems.00324-18-sf001.docx]

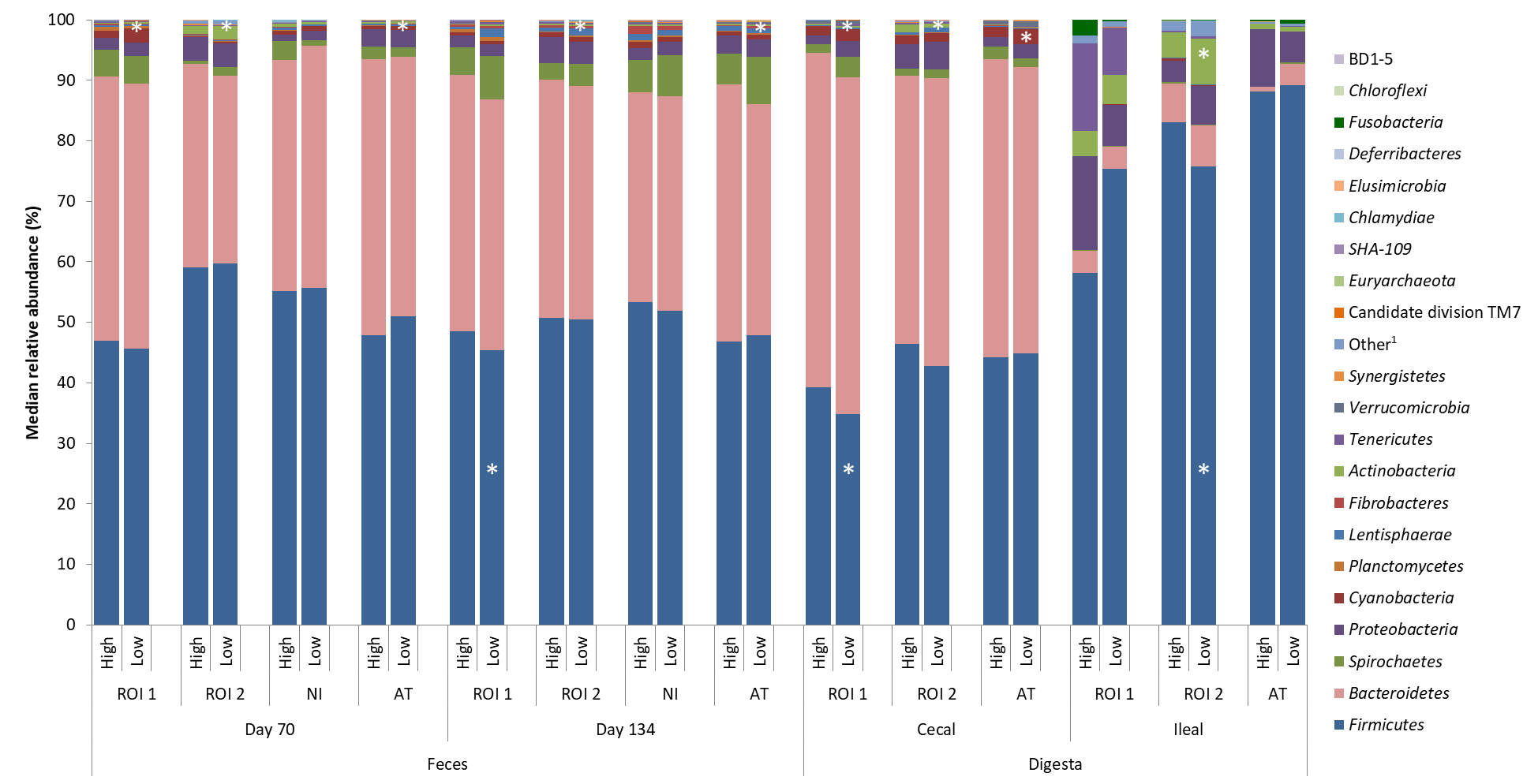

Supplement: FIG S2 [file mSystems.00324-18-sf002.docx]

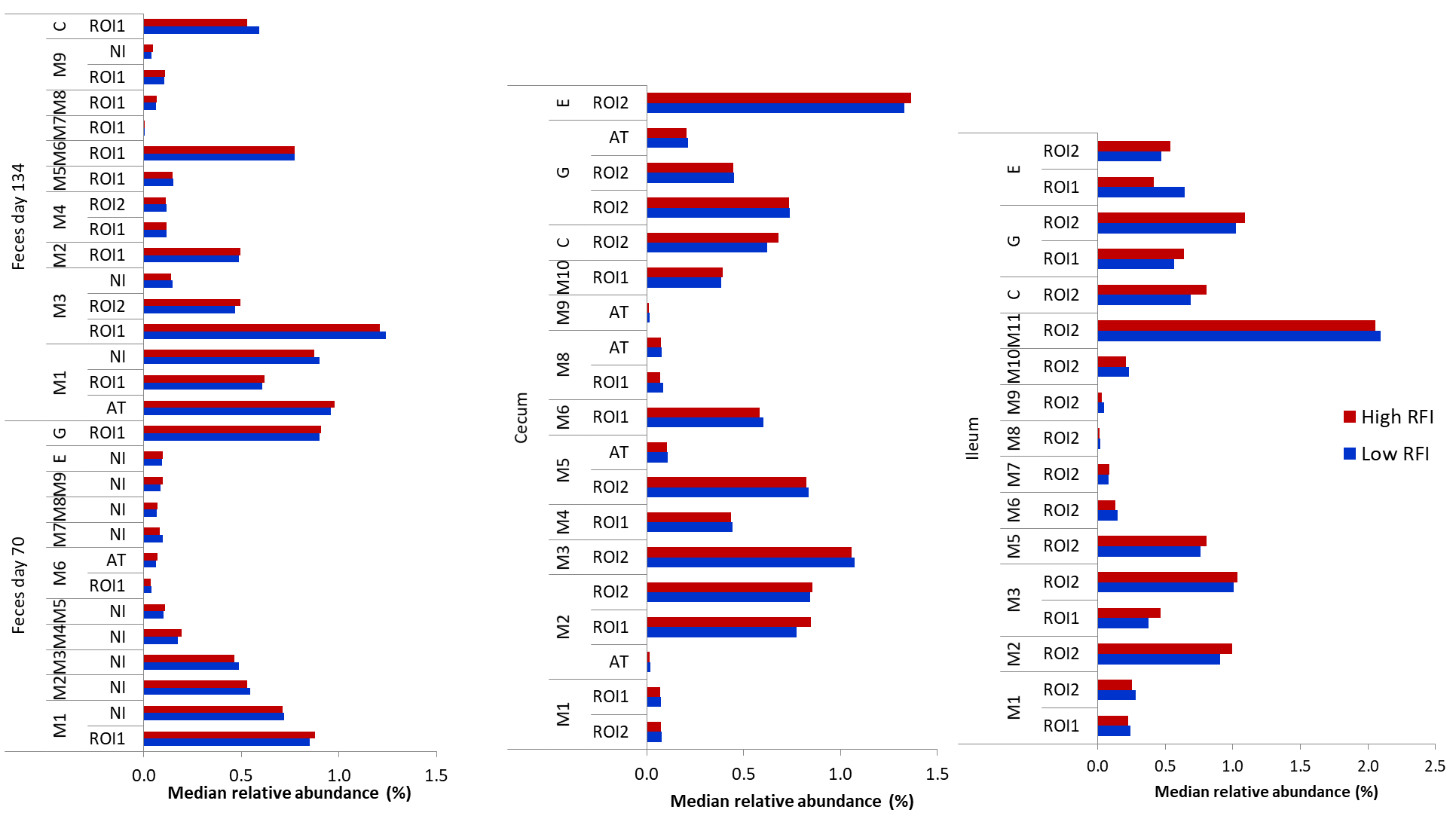


B. Digesta

A. Feces

Supplement: FIG S4 [file mSystems.00324-18-sf004.docx]
