# Supplementary material for: Porcine Feed Efficiency-Associated Intestinal Microbiota and Physiological Traits: Finding Consistent Cross-Locational Biomarkers for Residual Feed Intake
Source: mSystems. 2019 Jun 18;4(4):e00324-18. doi: 10.1128/mSystems.00324-18 (PMC6581691; doi:10.1128/mSystems.00324-18)
Supplement: FIG S3 [file mSystems.00324-18-sf003.docx]

**A. Feces at day 70**

**B. Feces at day 134**


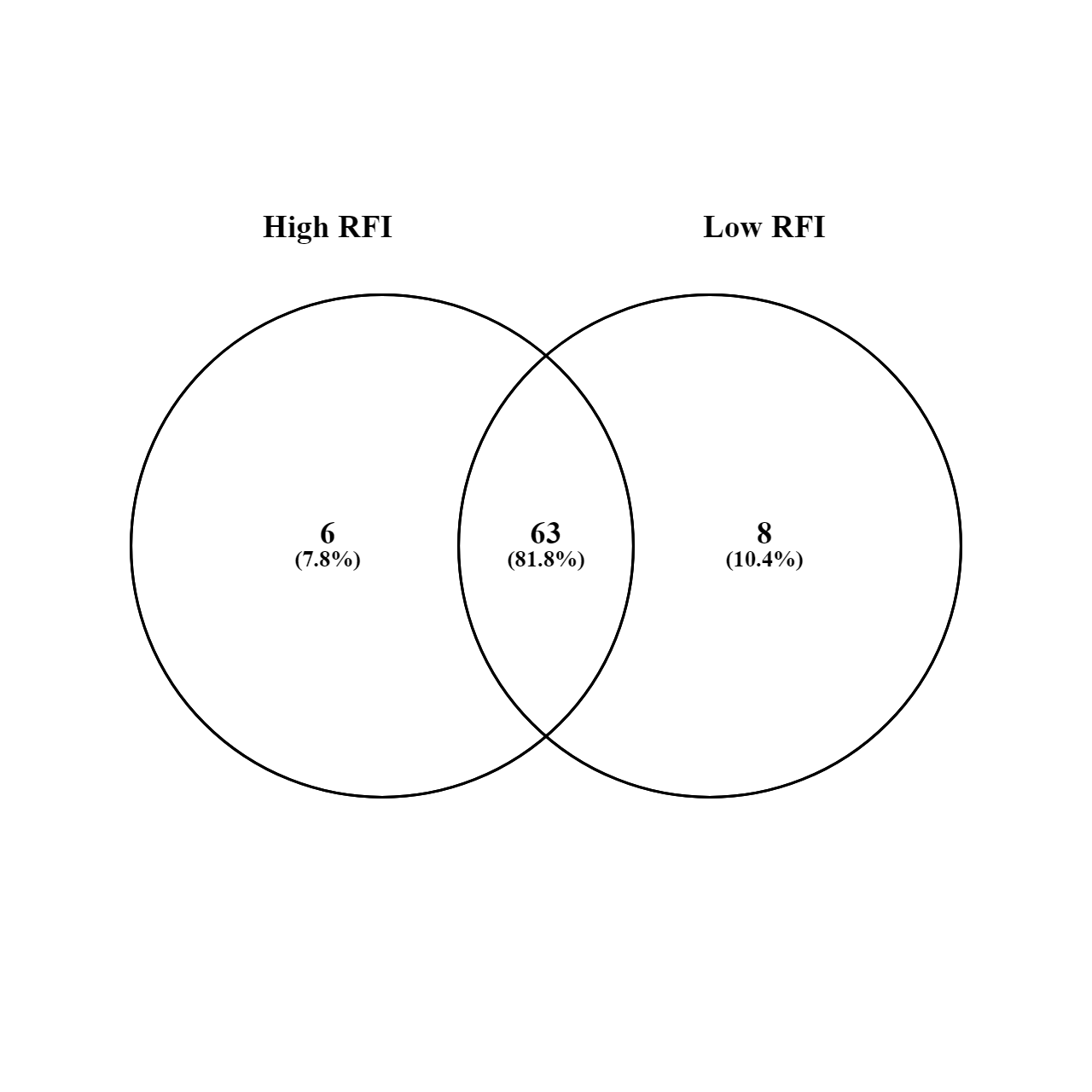


*Collinsella*

*Jeotgalicoccus*

*Enterococcus*

*Solobacterium*

*Megamonas*

*Helicobacter*

*Aeromonas*

*Actinobacillus*

8

(10.4%)

63

(81.8%)

6

(7.8%)

**High RFI**

**Low RFI**


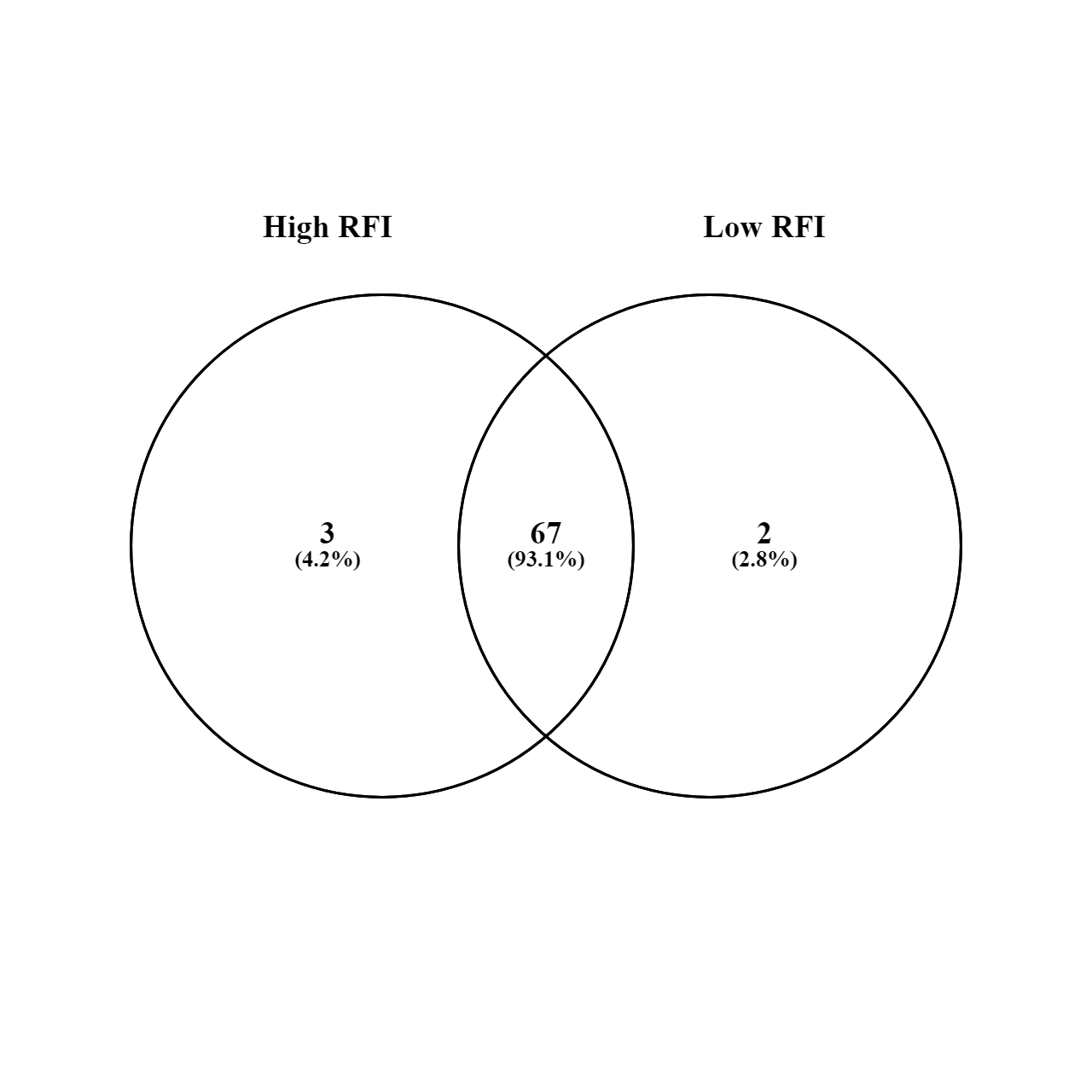


*Bacteroides*

*Verrucomicrobia subdivision 5 (unc)*

3

(4.2%)

67

(93.1%)

2

(2.8%)

**High RFI**

**Low RFI**


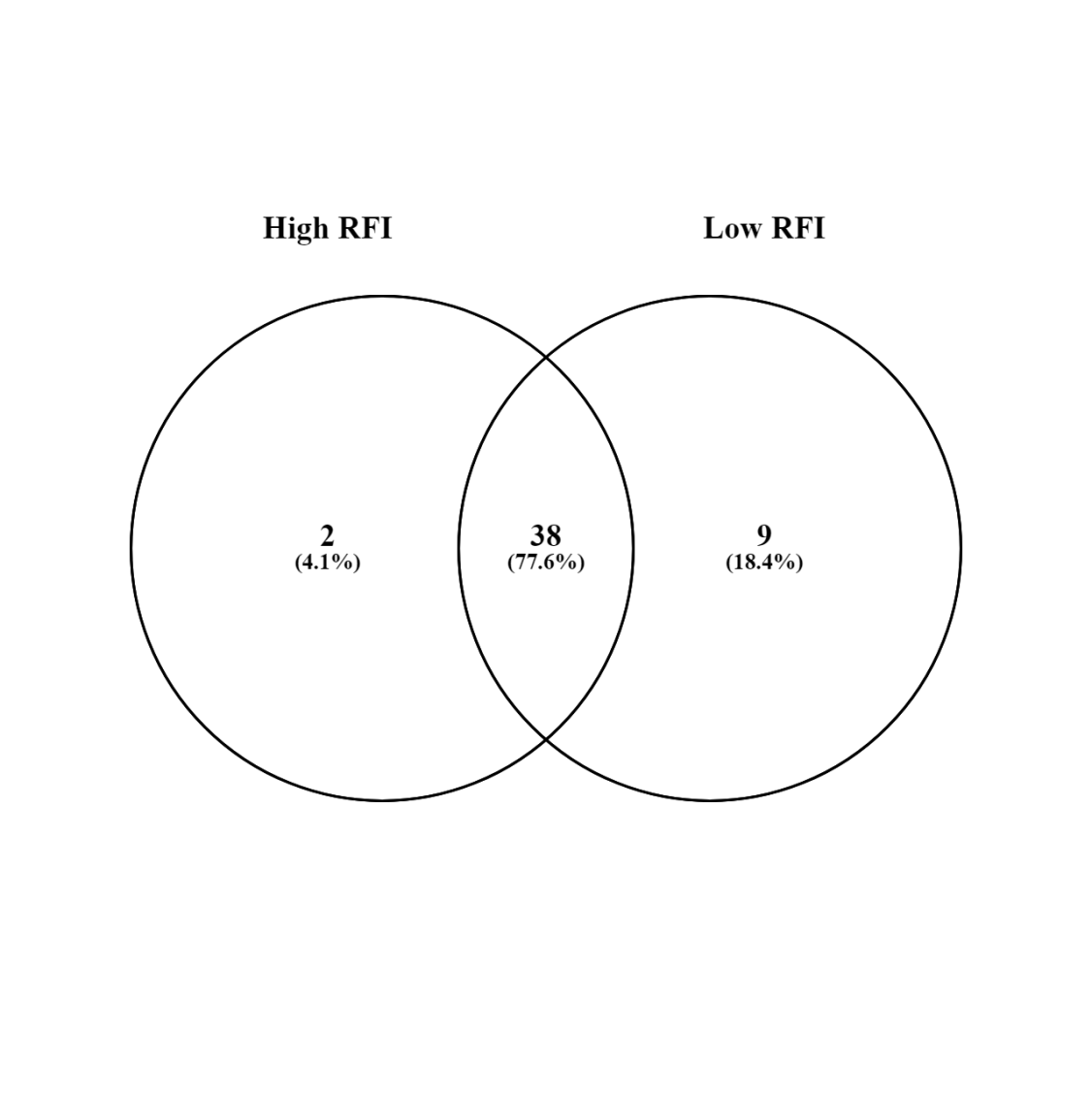


*Bifidobacterium*

*Brevibacterium*

*Brachybacterium*

*Sporosarcina*

*Salinicoccus*

*Staphylococcus*

*Sarcina*

*Erysipelotrichaceae (unc)*

*Aeromonas*

2

(4.1%)

38

(77.6%)

9

(18.4%)

**High RFI**

**Low RFI**


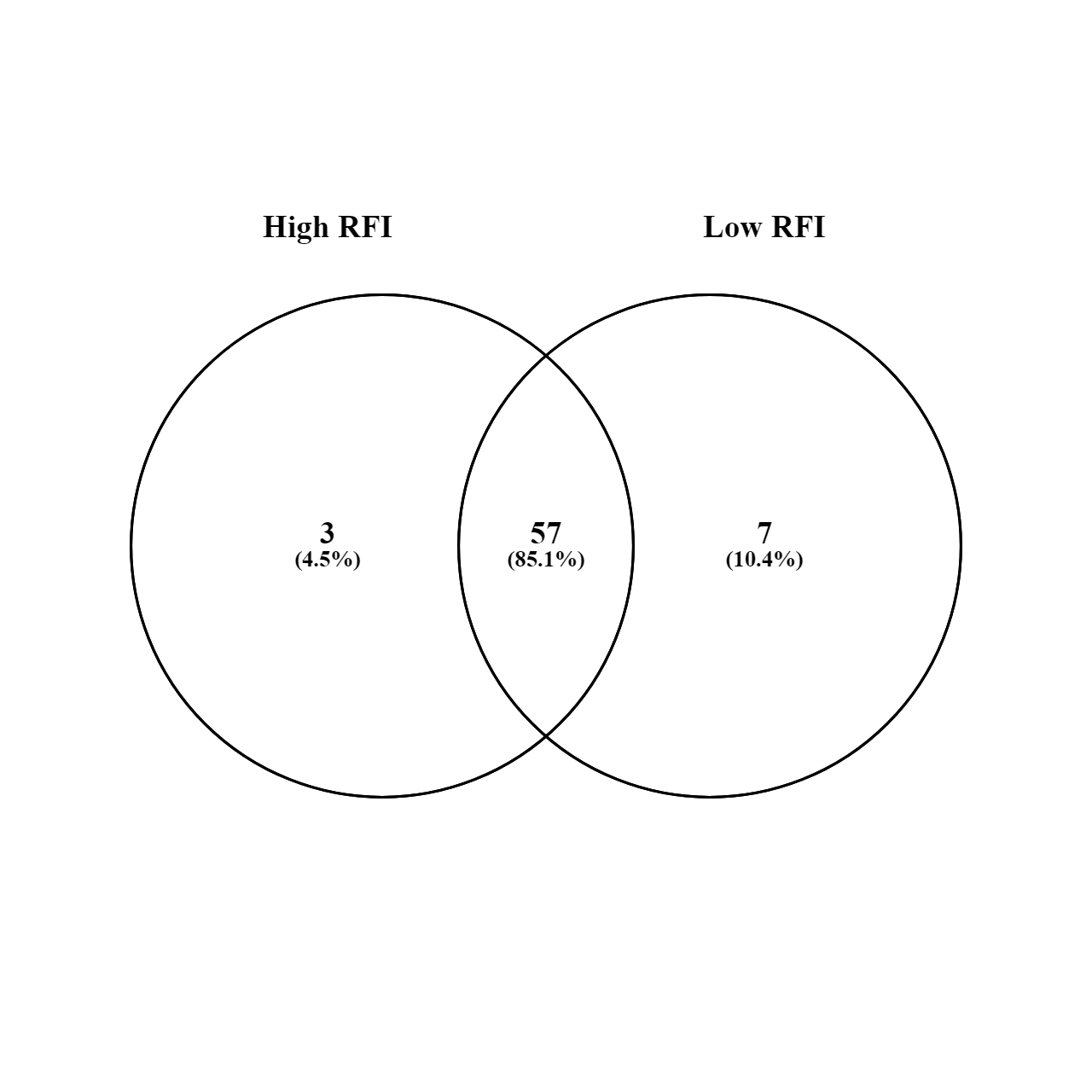


*Paludibacter*

*RFN43 (und)*

*dgA-11 gut group*

*Cellulosilyticum*

*vadinBB60 (unc)*

*Catenibacterium*

*Helicobacter*

3

(4.5%)

57

(85.1%)

7

(10.4%)

**High RFI**

**Low RFI**

**C. Ileal digesta**

**D. Cecal digesta**
