# Supplementary material for: Porcine Feed Efficiency-Associated Intestinal Microbiota and Physiological Traits: Finding Consistent Cross-Locational Biomarkers for Residual Feed Intake
Source: mSystems. 2019 Jun 18;4(4):e00324-18. doi: 10.1128/mSystems.00324-18 (PMC6581691; doi:10.1128/mSystems.00324-18)
Supplement: FIG S5 [file mSystems.00324-18-sf005.docx]

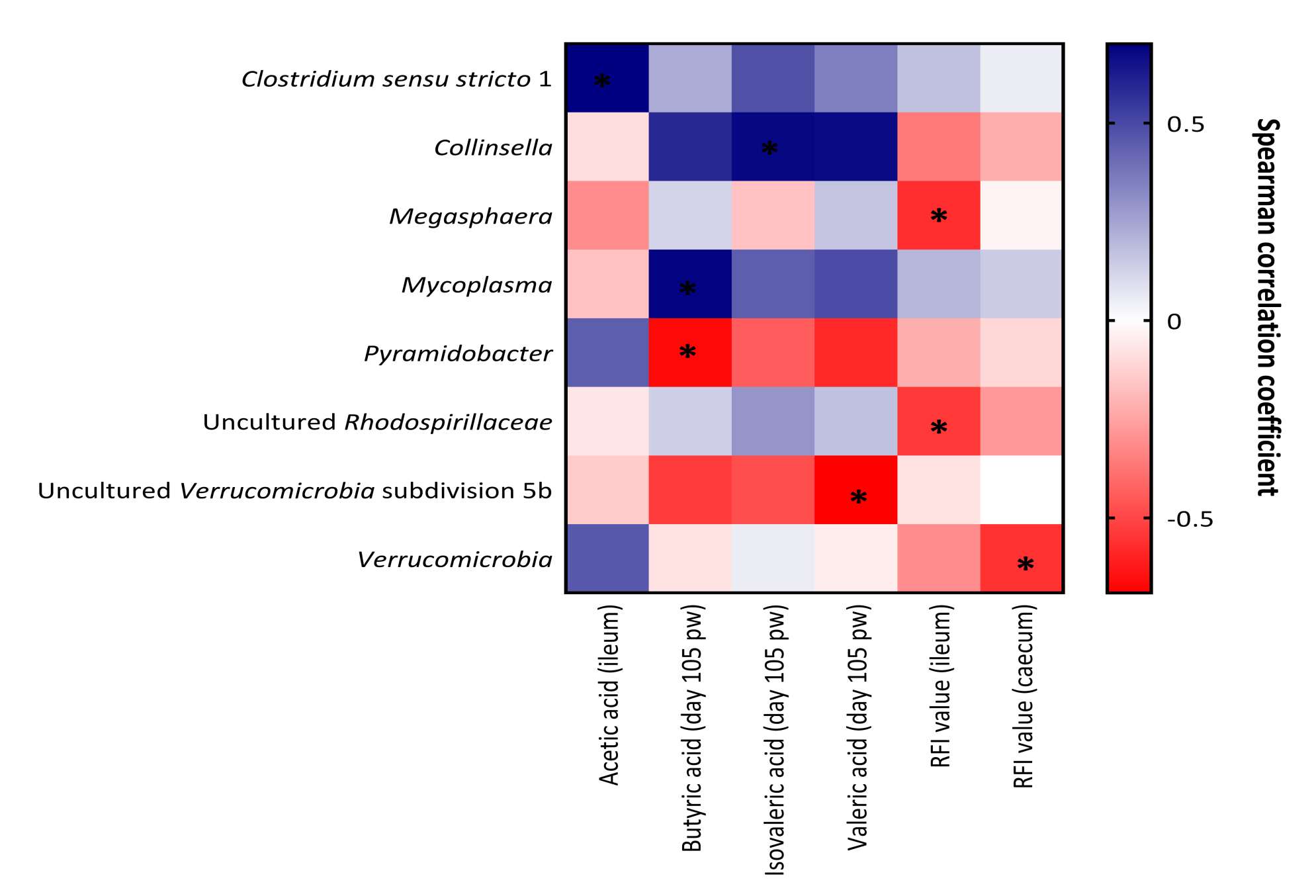


Acetic acid (Ileum)

Butyric acid (day 134)

Isovaleric acid (day 134)

Valeric acid (day 134)

RFI value (Ileum)

RFI value (Cecum)
