# Supplementary material for: Porcine Feed Efficiency-Associated Intestinal Microbiota and Physiological Traits: Finding Consistent Cross-Locational Biomarkers for Residual Feed Intake
Source: mSystems. 2019 Jun 18;4(4):e00324-18. doi: 10.1128/mSystems.00324-18 (PMC6581691; doi:10.1128/mSystems.00324-18)
Supplement: TABLE S1 [file mSystems.00324-18-st001.docx]

| **Measure** | **High RFI** | | | | **Low RFI** | | | |  | **SEM^4^** | **P-value** | | | | |
| --- | --- | --- | --- | --- | --- | --- | --- | --- | --- | --- | --- | --- | --- | --- | --- |
|  | **ROI1** | **ROI2** | **AT** |  | | **ROI1** | **ROI2** | **AT** |  |  | **Location* RFI** | **Location** | | | **RFI** |
| White blood cells (x 10^3^ cells/µl) | 27.6 | 25.4 | 16.1 |  | | 26.3 | 23.7 | 19.5 |  | 3.07 | 0.16 | 0.12 | | | 0.93 |
| Lymphocytes |  |  |  |  | |  |  |  |  |  |  |  | | |  |
| % | 34.2 | 45.1 | 52.7 |  | | 38.4 | 52.2 | 51.4 |  | 6.08 | 0.25 | 0.16 | | | 0.23 |
| no. x 10^3^ cells/µl | 9.8 | 12.3 | 6.6 |  | | 10.6 | 13.1 | 7.5 |  | 1.85 | 0.99 | 0.16 | | | 0.39 |
| Monocystes |  |  |  |  | |  |  |  |  |  |  |  | | |  |
| % | 10.2 | 15.8 | 10.0 |  | | 9.8 | 13.9 | 8.3 |  | 3.39 | 0.78 | 0.52 | | | 0.30 |
| no. x 10^3^ cells/µl | 2.53 | 3.87 | 2.50 |  | | 2.47 | 3.13 | 2.16 |  | 0.765 | 0.47 | 0.57 | | | 0.25 |
| Granulocytes |  |  |  |  | |  |  |  |  |  |  |  | | |  |
| % | 44.1 | 36.3 | - |  | | 45.7 | 36.4 | - |  | 7.15 | 0.89 | 0.45 | | | 0.87 |
| no. x 10^3^ cells/µl | 12.2 | 8.5 | - |  | | 11.7 | 8.1 | - |  | 2.41 | 0.97 | 0.35 | | | 0.81 |
| Red blood cells (x 10^6^ cells/µL) | 7.1 | 6.8 | 5.9 |  | | 7.1 | 6.7 | 6.1 |  | 0.59 | 0.85 | 0.41 | | | 0.90 |
| Red cell distribution width (fL) | 19.5 | 21.1 | 19.0 |  | | 19.9 | 21.2 | 19.1 |  | 2.05 | 0.95 | 0.81 | | | 0.73 |
| Haemoglobin (g/dL) | 14.1 | 12.5 | 11.7 |  | | 14.1 | 12.3 | 11.5 |  | 0.77 | 0.93 | 0.07 | | | 0.70 |
| Haematocrit (%) | 0.39 | 0.35 | 0.36 |  | | 0.36 | 0.39 | 0.36 |  | 0.022 | 0.66 | 0.35 | | | 0.97 |
| Mean corpuscular volume (fL) | 55.3 | 52.5 | 53.9 |  | | 56.5 | 54.7 | 53.9 |  | 1.47 | 0.45 | | 0.45 | 0.21 | |
| Mean corpuscular haemoglobin |  |  |  |  | |  |  |  |  |  |  | |  |  | |
| % | 18.4 | 16.6 | 16.5 |  | | 18.4 | 16.9 | 16.4 |  | 0.49 | 0.76 | | 0.01 | 0.76 | |
| pg | 33.1 | 31.8 | 28.7 |  | | 33.2 | 32.2 | 29.7 |  | 0.89 | 0.63 | | 0.01 | 0.29 | |
| Platelets (x 10^6^ cells /µL) | 226 | 286 | 147 |  | | 207 | 238 | 145 |  | 45.0 | 0.61 | | 0.24 | 0.38 | |
| Mean platelet volume (fL) | 9.4 | 9.7 | 7.7 |  | | 9.2 | 9.0 | 7.8 |  | 1.05 | 0.80 | | 0.70 | 0.72 | |
|  |  |  |  |  | |  |  |  |  |  |  | |  |  | |
| Creatinine (µmol/L) | 133.4 | 111.1 | - |  | | 139.2 | 117.9 | - |  | 8.66 | 0.17 | | 0.07 | 0.91 | |
| Creatine kinase (µmol/L) | 113.1 | 96.5 | - |  | | 136.4 | 81.8 | - |  | 15.57 | 0.78 | | 0.02 | 0.28 | |
| Total protein (g/L) | 66.5 | 55.1 | - |  | | 66.9 | 59.7 | - |  | 4.70 | 0.71 | | 0.19 | 0.49 | |
| Triglycerides (mmol/L) | 0.61 | 0.53 | 0.36 |  | | 0.57 | 0.56 | 0.31 |  | 0.061 | 0.57 | | 0.009 | 0.49 | |
| Glucose (mmol/L) | 5.39 | 4.53 | 6.41 |  | | 5.82 | 4.75 | 6.48 |  | 0.358 | 0.23 | | 0.001 | 0.80 | |
| Cholesterol (mmol/L) | 2.57 | 2.08 | 2.46 |  | | 2.53 | 2.28 | 2.35 |  | 0.194 | 0.85 | | 0.30 | 0.27 | |
| Blood urea nitrogen (mg/dL) | 15.3 | 12.2 | 17.4 |  | | 14.9 | 11.9 | 15.6 |  | 1.87 | 0.89 | | 0.06 | 0.58 | |

^1^Blood was collected at slaughter (day 134 days of age); ^2^ROI: Republic of Ireland; ^3^AT: Austria; ^4^ Least squares means and the pooled standard error of the mean are presented.
