# Supplementary material for: Porcine Feed Efficiency-Associated Intestinal Microbiota and Physiological Traits: Finding Consistent Cross-Locational Biomarkers for Residual Feed Intake
Source: mSystems. 2019 Jun 18;4(4):e00324-18. doi: 10.1128/mSystems.00324-18 (PMC6581691; doi:10.1128/mSystems.00324-18)
Supplement: TABLE S2 [file mSystems.00324-18-st002.docx]

| **Measure** | | **High RFI** | | | **Low RFI** | | | **SEM^2^** | | **P-value** |
| --- | --- | --- | --- | --- | --- | --- | --- | --- | --- | --- |
| ***Pooled ileal LPL and IEL cells*** | | | | | | | | | | |
| ***Control (PBS)*** | | | | | | | | | | |
| B cells | 1.74 | | 1.79 | | | 0.484 | | | 0.99 | |
| T cells | 2.49 | | 1.82 | | | 0.589 | | | 0.86 | |
| CD4 T cells | 80.9 | | 87.5 | | | 8.39 | | | 0.95 | |
| CD8 T cells | 6.36 | | 5.81 | | | 1.129 | | | 0.98 | |
| CD4CD8 T cells | 6.26 | | 6.41 | | | 2.433 | | | 0.99 | |
| γδ T cells | 0.97 | | 0.82 | | | 0.156 | | | 0.91 | |
| Monocytes | 7.94 | | 7.66 | | | 2.282 | | | 0.99 | |
|  | | | | | | | | | | |
| ***Mitogen-stimulated (PMA+I)^3^*** | | | | | | | | | | |
| B cells | 1.86 | | 2.04 | | | 0.485 | | | 0.99 | |
| T cells | 3.86 | | 2.52 | | | 0.590 | | | 0.39 | |
| CD4 T cells | 70.3 | | 81.9 | | | 8.40 | | | 0.76 | |
| CD8 T cells | 5.71 | | 4.79 | | | 1.130 | | | 0.93 | |
| CD4CD8 T cells | 4.78 | | 11.02 | | | 2.432 | | | 0.18 | |
| γδ T cells | 0.62 | | 0.64 | | | 0.157 | | | 0.99 | |
| Monocytes | 6.77 | | 4.47 | | | 2.282 | | | 0.89 | |
|  | | | | | | | | | | |
| ***Cytokine production from pooled ileal LPL and IEL*** | | | | | | | | | | |
| ***Control (PBS)*** | | | | | | | | | | |
| IL-4 | | 3.28 | | 4.94 | | | 0.260 | | 0.69 | |
| IL-6 | | 3.77 | | 2.41 | | | 0.310 | | 0.76 | |
| IL-8 | | 7.76 | | 5.61 | | | 0.403 | | 0.61 | |
| TNFα | | 3.28 | | 3.92 | | | 0.302 | | 0.98 | |
|  | |  | |  | | |  | |  | |
| ***Mitogen-stimulated (PMA+I)*** | | | | | | | | | | |
| IL-4 | | 4.16 | | 3.86 | | | 0.270 | | 0.97 | |
| IL-6 | | 4.36 | | 4.08 | | | 0.320 | | 0.99 | |
| IL-8 | | 5.93 | | 6.33 | | | 0.400 | | 0.71 | |
| TNFα | | 2.58 | | 3.73 | | | 0.300 | | 0.83 | |

^1^Percentages are based on total white blood cells.

^2^Least squares means and the pooled standard error of the mean are presented.

^3^PMA+I: pooled IEL and LPL were mitogen-stimulated with phorbol myristate acetate (PMA, 25 ng/mL) plus ionomycin (I; 1 μg/mL).
