# Supplementary material for: Porcine Feed Efficiency-Associated Intestinal Microbiota and Physiological Traits: Finding Consistent Cross-Locational Biomarkers for Residual Feed Intake
Source: mSystems. 2019 Jun 18;4(4):e00324-18. doi: 10.1128/mSystems.00324-18 (PMC6581691; doi:10.1128/mSystems.00324-18)
Supplement: TABLE S3 [file mSystems.00324-18-st003.docx]

| Measure | High RFI | | | | Low RFI | | | | S.E.M | P-value |
| --- | --- | --- | --- | --- | --- | --- | --- | --- | --- | --- |
|  | ROI1^3^ | ROI2 | NI^4^ | AT^5^ | ROI1 | ROI2 | NI | AT |  |  |
| Log EU^6^/g  (Fresh matter) | 6.45 | 6.09 | 6.07 | 6.16 | 6.17 | 6.01 | 6.02 | 6.11 | 0.065 | 0.76 |
| Log EU/g  (Dry matter) | 7.03 | 6.97 | 7.05 | 7.11 | 7.06 | 6.92 | 7.05 | 7.10 | 0.062 | 0.86 |
|  |  |  |  |  |  |  |  |  |  |  |
| Serum haptoglobin (µg/ml) | 172.1 | - | 171.4 | 180.8 | 155.8 | - | 186.6 | 180.4 | 35.74 | 0.47 |

^1^ caecal lipopolysaccharides were measured in digesta collected at slaughter (~134 days of age), ^2^ haptoglobin concentrations were measured in serum collected from pigs in ^3^ ROI: Republic of Ireland (batch 1 only), ^4^ NI: Northern Ireland, and ^5^ AT: Austria. ^6^ EU: endotoxin units.

Least square means and pooled standard error of the mean are presented.
