# Supplementary material for: Porcine Feed Efficiency-Associated Intestinal Microbiota and Physiological Traits: Finding Consistent Cross-Locational Biomarkers for Residual Feed Intake
Source: mSystems. 2019 Jun 18;4(4):e00324-18. doi: 10.1128/mSystems.00324-18 (PMC6581691; doi:10.1128/mSystems.00324-18)
Supplement: TABLE S4 [file mSystems.00324-18-st004.docx]

| **Ingredient** | **Starter** | **Link** | **Weaner** | **Finisher** | **Pregnant sow** | **Lactating sow** |
| --- | --- | --- | --- | --- | --- | --- |
| Barley |  |  | 248.0 | 385.4 | 897.4 | 349.5 |
| Wheat | 220.0 | 399.0 | 431.4 | 404.0 |  | 432.4 |
| Maize | 80.0 |  |  |  |  |  |
| Soya | 163.5 | 229.2 | 200.0 | 175.0 | 70.0 | 150.0 |
| Full fat soya | 100.0 | 70.0 | 50.0 |  |  |  |
| Lactofeed 70^1^ | 200.0 | 200.0 |  |  |  |  |
| Skim milk powder | 125.0 | 50.0 |  |  |  |  |
| Soya oil | 78.1 | 25.0 | 40.0 | 10.0 | 10.0 | 40.0 |
| Lysine HCl (78.8) | 4.73 | 3.70 | 4.6 | 4.0 | 1.0 | 3.5 |
| DL-Methionine | 3.22 | 2.33 | 1.70 | 1.00 |  | 1.00 |
| L-Threonine | 2.41 | 1.62 | 2.00 | 1.50 |  | 1.00 |
| L-Tryptophan | 0.95 | 0.54 | 0.20 | 0.00 |  |  |
| Vitamins and minerals | 3.0^2^ | 3.0^2^ | 3.0^2^ | 1.0^3^ | 1.5^4^ | 1.5^4^ |
| Natuphos 5000 FTU/g^5^ | 0.10 | 0.10 | 0.10 | 0.10 | 0.10 | 0.10 |
| Salt | 3.0 | 3.0 | 3.0 | 3.0 | 4.0 | 4.0 |
| Dicalcium phosphate | 5.00 | 1.52 | 5.00 | 2.00 | 5.00 | 5.00 |
| Limestone flour | 11.0 | 11.0 | 11.0 | 13.0 | 11.0 | 12.0 |
|  |  |  |  |  |  |  |
| **Chemical analysis (g/kg dry matter)** | | |  |  |  |  |
| Crude protein | 235.9 | 252.8 | 211.0 | 205.4 | 195.7 | 172.1 |
| Crude fibre | 19.8 | 23.4 | 34.8 | 37.5 | 38.9 | 31.7 |
| Crude ash | 66.2 | 63.5 | 48.3 | 45.4 | 44.6 | 49.8 |
| Ether extract | 114.7 | 57.9 | 70.7 | 27.2 | 33.2 | 63.4 |
| Digestible energy (MJ/kg)^6^ | 17.9 | 17.0 | 16.9 | 16.0 | 15.9 | 16.3 |
| Net energy ((MJ/kg) | 11.4 | 10.3 | 10.6 | 9.8 | 9.5 | 10.5 |
|  |  |  |  |  |  |  |
| **Amino acids (g/kg)** | |  |  |  |  |  |
| Lysine | 16.2 | 15.0 | 13.0 | 11.1 | 6.4 | 9.9 |
| Methionine | 6.8 | 5.7 | 4.5 | 3.6 | 2.1 | 3.4 |
| Methionine + cysteine | 9.7 | 9.0 | 7.9 | 6.8 | 4.7 | 6.4 |
| Threonine | 10.5 | 9.8 | 8.7 | 7.5 | 4.5 | 6.5 |
| Tryptophan | 3.6 | 3.3 | 2.6 | 2.2 | 1.6 | 2.0 |

^1^Lactofeed 70: 70% lactose, 11.5% protein, 7.5% ash, 0.5% oil, and 0.5% fibre (Volac, Cambridge, UK)

^2^Premix provided per kg of complete diet: Cu 155mg, FE 90mg, Mn 47mg, Zn 120mg, I 0.6mg, Se 0.3mg, Vit A 6000 IU, Vit D_3_ 1000 IU, Vit E 100 IU, Vit K 4mg, Vit B_12_ 15µg, Riboflavin 2mg, Nicotinic acid 12mg, Pantothenic acid 10mg, Choline chloride 250mg, Vit B_1_ 2mg, Vit B_6_ 3mg, Endox 60g.

^3^Premix provided per kg of complete diet: Cu 15mg, FE 24mg, Mn 31mg, Zn 80mg, I 0.3mg, Se 0.2mg, Vit A 2000 IU, Vit D_3_ 500 IU, Vit E 40 IU, Vit K 4mg, Vit B_12_ 15µg, Riboflavin 2mg, Nicotinic acid 12mg, Pantothenic acid 10mg, Vit B_1_ 2mg, Vit B_6_ 3mg.

^4^Premix provided per kg of complete diet: Cu 15mg, FE 70mg, Mn 62mg, Zn 80mg, I 0.6mg, Se 0.2mg, Vit A 1000 IU, Vit D_3_ 1000 IU, Vit E 100 IU, Vit K 2mg, Vit B_12_ 15µg, Riboflavin 5mg, Nicotinic acid 12mg, Pantothenic acid 10mg, Choline chloride 500mg, Biotin 200mg, Folic acid 5g, Vit B_1_ 2mg, Vit B_6_ 3mg.

^5^Phytase: 5000 FTU/g = 50 FTU per kg finished feed.

^6^Digestible and net energy were calculated from book values.

Diets were pelleted to 3mm diameter after steam conditioning to 50-55°C.
